# Molecular Determinants and Genetic Modifiers of Aggregation and Toxicity for the ALS Disease Protein FUS/TLS

**DOI:** 10.1371/journal.pbio.1000614

**Published:** 2011-04-26

**Authors:** Zhihui Sun, Zamia Diaz, Xiaodong Fang, Michael P. Hart, Alessandra Chesi, James Shorter, Aaron D. Gitler

**Affiliations:** 1 Department of Cell and Developmental Biology, The University of Pennsylvania School of Medicine, Philadelphia, Pennsylvania, United States of America; 2 Department of Biochemistry and Biophysics, The University of Pennsylvania School of Medicine, Philadelphia, Pennsylvania, United States of America; University of California San Francisco/Howard Hughes Medical Institute, United States of America

## Abstract

TDP-43 and FUS are RNA-binding proteins that form cytoplasmic inclusions in some forms of amyotrophic lateral sclerosis (ALS) and frontotemporal lobar degeneration (FTLD). Moreover, mutations in TDP-43 and FUS are linked to ALS and FTLD. However, it is unknown whether TDP-43 and FUS aggregate and cause toxicity by similar mechanisms. Here, we exploit a yeast model and purified FUS to elucidate mechanisms of FUS aggregation and toxicity. Like TDP-43, FUS must aggregate in the cytoplasm and bind RNA to confer toxicity in yeast. These cytoplasmic FUS aggregates partition to stress granule compartments just as they do in ALS patients. Importantly, in isolation, FUS spontaneously forms pore-like oligomers and filamentous structures reminiscent of FUS inclusions in ALS patients. FUS aggregation and toxicity requires a prion-like domain, but unlike TDP-43, additional determinants within a RGG domain are critical for FUS aggregation and toxicity. In further distinction to TDP-43, ALS-linked FUS mutations do not promote aggregation. Finally, genome-wide screens uncovered stress granule assembly and RNA metabolism genes that modify FUS toxicity but not TDP-43 toxicity. Our findings suggest that TDP-43 and FUS, though similar RNA-binding proteins, aggregate and confer disease phenotypes via distinct mechanisms. These differences will likely have important therapeutic implications.

## Abstract 2

A combination of yeast genetics and protein biochemistry define how the fused in sarcoma (FUS) protein might contribute to Lou Gehrig's disease.

## Author Summary

Many human neurodegenerative diseases are associated with the abnormal accumulation of protein aggregates in the neurons of affected individuals. Amyotrophic lateral sclerosis (ALS), also known as Lou Gehrig's disease, is a fatal human neurodegenerative disease caused primarily by a loss of motor neurons. Recently, mutations in a gene called fused in sarcoma (FUS) were identified in some ALS patients. The basic mechanisms by which FUS contributes to ALS are unknown. We have addressed this question using protein biochemistry and the genetically tractable yeast *Saccharomyces cerevisiae.* We defined the regions of biochemically pure FUS protein that contribute to its aggregation and toxic properties. We then used genome-wide screens in yeast to identify genes and cellular pathways involved in the toxicity of FUS. Many of the FUS toxicity modifier genes that we identified in yeast have clear homologs in humans, suggesting that these might also be relevant for the human disease. Together, our studies provide novel insight into the basic mechanisms associated with FUS aggregation and toxicity. Moreover, our findings open new avenues that could be explored for therapeutic intervention.

## Introduction

Amyotrophic lateral sclerosis (ALS), also called Lou Gehrig's disease, is a devastating neurodegenerative disease. It is a rapidly progressing motor neuron wasting disorder that leads to paralysis and death typically within 2–5 years of onset. There are no cures or effective treatments. Given the similarities in presentation and pathology of familial and sporadic disease, study of genes mutated in familial disease can shed light on mechanisms of both familial ALS and the more common sporadic form. The first familial gene associated with ALS was *SOD1* [1], and much research over the past 10–15 years has focused on mechanisms by which mutant SOD1 may cause motor neuron dysfunction and loss [2].

Insight into ALS changed dramatically in 2006 when the 43 kDa TAR-DNA-binding protein (TDP-43) was identified as a protein that accumulates abnormally in the ubiquitinated pathological lesions that characterize brain and spinal cord tissue of almost every non-*SOD1* ALS patient [3]–[5]. Similar TDP-43 inclusions were also identified in degenerating neurons in a subset of frontotemporal lobar degeneration (FTLD-TDP) cases [3]–[5]. TDP-43 is an RNA-binding protein with two RNA recognition motifs (RRMs) and a glycine rich domain [6]. In 2008, several groups independently reported the identification of over 30 different mutations in the TDP-43 gene (*TARDBP*) in various sporadic and familial ALS patients [6]–[10]. TDP-43 mutations were subsequently identified in various FTLD-TDP cases [11],[12]. Taken together, these studies strongly suggest that TDP-43 is a new human neurodegenerative disease protein. Wild-type (WT) TDP-43 accumulates abnormally in cytoplasmic, ubiquitinated inclusions in degenerating neurons of ALS and FTLD-TDP patients, and mutations in the TDP-43 gene are linked with disease in rare familial and sporadic cases. Despite these advances, how TDP-43 contributes to disease, which domain of TDP-43 drives aggregation, and how ALS-linked mutations affect TDP-43 function and aggregation remained unclear.

**Figure 1:**
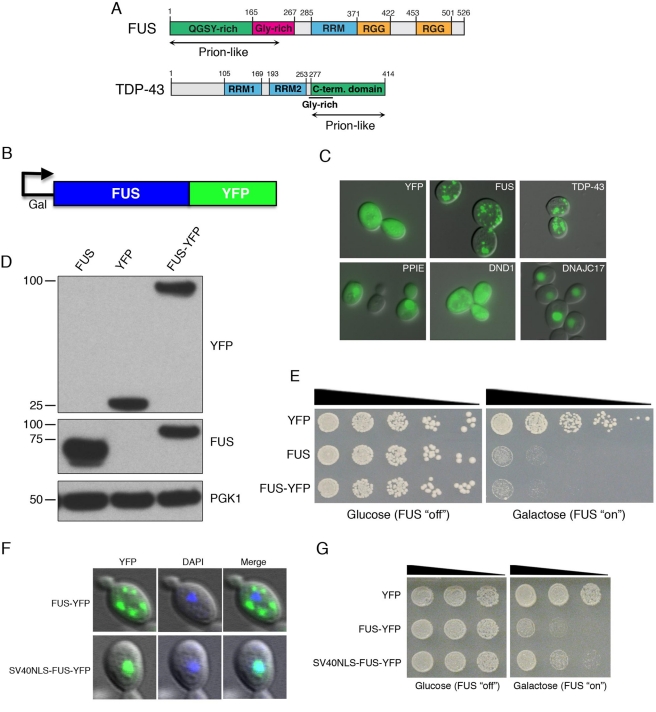
Yeast FUS model. (A) Schematic of domain architecture of FUS and TDP-43. Note that both contain glycine-rich regions, RRM, and prion-like domains. In addition, FUS has two RGG domains. (B) Schematic of galactose-inducible construct to express human FUS fused to YFP. (C) Yeast cells expressing YFP alone or YFP-tagged human RNA-binding proteins. Not all RNA-binding proteins aggregate when expressed in yeast. For example, three related human RRM-containing proteins did not form inclusions when expressed in yeast. Instead they were diffusely localized: PPIE localized to the nucleus and cytoplasm; DND1 localized to the cytoplasm; and DNAJC17 was restricted to the nucleus. The ALS disease proteins, TDP-43 and FUS, formed multiple cytoplasmic foci when expressed in yeast. (D) Immunoblot showing untagged and YFP-tagged FUS expression. (E) FUS is toxic when expressed in yeast cells compared to YFP alone control. 5-fold serial dilutions of yeast cells expressing YFP alone, untagged FUS, or YFP-tagged FUS. Because of the galactose-inducible promoter, FUS expression is repressed when cells are grown in the presence of glucose (left panel, FUS expression “off”) and induced when grown in the presence of galactose (right panel, FUS expression “on”). (F) Fusing the strong heterologous SV40 NLS to the N-terminus of FUS restricts it mostly to the nucleus. (G) Spotting assay shows that nuclear-localized SV40 FUS-YFP is less toxic than WT FUS.

To address these deficits, we investigated the pathogenic properties of TDP-43 in yeast. The yeast system is simple and fast and has highly conserved fundamental pathways that allow powerful insights into complex human neurodegenerative diseases such as Parkinson's disease, Alzheimer's disease, and ALS [13]. Therefore, we developed a yeast model of TDP-43 to study TDP-43 biology as well as the mechanisms of TDP-43 aggregation and toxicity. Expression of human TDP-43 in yeast resulted in cytoplasmic aggregation and toxicity, thus modeling key aspects of human TDP-43 proteinopathies. These studies revealed that RRM2 and the C-terminal domain of TDP-43 (Figure 1A) are required for aggregation and toxicity [14]. Notably, all but one of over 30 ALS-linked mutations reside in the C-terminal domain, which the yeast system defined as critical for toxicity. Moreover, a combination of pure protein studies and in vivo analyses in yeast demonstrated that ALS-linked TDP-43 mutations render TDP-43 more aggregation-prone and enhance toxicity [15]. These studies demonstrated that the aggregation propensity and severity of toxicity of TDP-43 variants observed in ALS could be recapitulated in yeast. Moreover, we have discovered a potent genetic modifier of TDP-43 toxicity in yeast, Pbp1, which is connected with ALS in humans [16]. The human homolog of Pbp1, ataxin 2, harbors a polyglutamine tract that is greatly expanded (>34 glutamines) in spinocerebellar ataxia type 2 [16]. Importantly, intermediate-length polyQ expansions (∼27–33 glutamines) in ataxin 2 are a significant genetic risk factor for ALS in humans [16]. Clearly, the power of yeast genetics can be exploited to define basic disease mechanisms of fundamental importance to human neurodegenerative disease.

Shortly following the identification of mutations in TDP-43 in ALS, mutations in another gene encoding an RNA-binding protein, FUS (fused in sarcoma; also known as TLS, translocated in liposarcoma), were connected to familial ALS [17],[18]. Additional mutations in FUS have recently been identified in sporadic ALS cases and in a subset of frontotemporal lobar degeneration (FTLD-FUS) cases [19],[20]. FUS is normally a nuclear protein, but ALS patients harboring FUS mutations exhibit prominent neuronal cytoplasmic FUS accumulations that appear devoid of TDP-43 [18]. Several other examples of neurodegenerative disease are beginning to emerge where the predominant disease phenotype is the cytoplasmic aggregation of wild-type FUS. These include some cases of juvenile ALS [21], basophilic inclusion body disease [22], as well as the majority of tau- and TDP-43-negative frontotemporal lobar degeneration cases [23]. Moreover, FUS is also aggregated in Huntington's disease; spinocerebellar ataxia (SCA) type 1, 2, and 3; and dentatorubropallidoluysian atrophy [24],. These findings extend the spectrum of disorders associated with FUS aggregation beyond ALS and FTLD-FUS and suggest the importance of understanding mechanisms of aggregation of WT as well as mutant FUS.

FUS was initially discovered as part of a chromosomal translocation associated with human myxoid liposarcomas [26]. Subsequent studies have revealed roles for FUS in transcription, RNA processing, and RNA transport [27]–[29]. In neurons, FUS is localized to the nucleus but is transported to dendritic spines at excitatory post-synapses in a complex with RNA and other RNA-binding proteins [30]. In further support of a role of FUS in maintaining neuronal architecture, primary hippocampal neurons cultured from FUS knockout mouse embryos display defects in spine morphology and decreased spine density [31]. It remains unclear, however, how loss of this function of FUS or perhaps a novel toxic gain-of-function associated with FUS mutations contribute to ALS. Importantly, it is also uncertain whether FUS is intrinsically aggregation-prone. Indeed, FUS might simply be a marker of disease that is sequestered by other aggregated components.

FUS and TDP-43 possess a similar domain structure. Like TDP-43, FUS has an RRM and a glycine-rich domain (Figure 1A). Moreover, using a bioinformatic algorithm designed to identify yeast prion domains [32], we recently identified novel “prion-like” domains in the N-terminal domain of FUS (amino acids 1–239) and in the C-terminal domain of TDP-43 (amino acids 277–414) (Figure 1A, Figure S1) [33]. Similar to prion domains found in yeast prion proteins such as Sup35, Ure2, and Rnq1, this domain is enriched in uncharged polar amino acids (especially asparagine, glutamine, and tyrosine) and glycine [32],[34]. This type of domain encodes all the information necessary to form a prion in yeast [27]–. It should be noted, however, that this type of domain is not found in all prion proteins, including HET-s from *Podospora anserina* and mammalian prion protein (PrP) [33],[34]. Remarkably, by using this bioinformatic algorithm [32] to score and rank the human proteome (27,879 human proteins) for prion-like properties, FUS and TDP-43 ranked 15^th^ and 69^th^, respectively [33]. Our findings raise the intriguing possibility that RRM proteins with predicted prion-like domains may be particularly relevant to ALS [15],[33],[35],[36]. Virtually all the ALS-linked mutations in TDP-43 lie in its prion-like domain [33]. By contrast, only a few of the ALS-linked mutations in FUS lie in its prion-like domain [33]. Indeed, the majority of ALS-linked FUS mutations reside at the extreme C-terminal region [37]. The identification of two RNA-binding proteins with a similar domain architecture that aggregate and are sometimes mutated in ALS and FTLD gives rise to the emerging concept that RNA metabolic pathways may play a major role in ALS and FTLD pathogenesis [38].

Despite these similarities between TDP-43 and FUS, it is unknown whether TDP-43 and FUS aggregate and cause toxicity by similar mechanisms. Here, we address this issue and establish, for the first time, two vital weapons in the fight against FUS proteinopathies, which have been critical in advancing our basic understanding of various other protein misfolding disorders, including Parkinson's disease, Huntington's disease, and TDP-43 proteinopathies [13]–[16],[39]–[45]. First, we establish a simple yeast model of FUS aggregation and toxicity. Second, we reconstitute FUS misfolding and aggregation using pure protein. These two approaches have served as important foundations for understanding mechanistic aspects of numerous neurodegenerative disorders and have empowered countless advances. We establish that, as for TDP-43, the RRM and the prion-like domain of FUS are required for aggregation and toxicity in yeast. However, in contrast to TDP-43, we find that additional determinants within the first RGG domain (Figure 1A) are also critical for FUS aggregation and toxicity. Importantly, we demonstrate that pure FUS is inherently aggregation-prone in the absence of other components and this behavior requires determinants in the prion-like domain and first RGG domain of FUS (Figure 1A). Aggregates formed by pure FUS are filamentous and resemble those formed by FUS in degenerating motor neurons of ALS patients. ALS-linked TDP-43 mutations can promote aggregation in vitro with pure proteins and in yeast [15]. By contrast, we find that ALS-linked FUS mutations do not promote aggregation per se. Finally, using two genome-wide screens in yeast, we identified several genes and pathways as potent modifiers of FUS toxicity. Many of the genes that we discovered in the yeast screens have human homologs. Thus, they are likely to provide insight into the specific cellular pathways perturbed by FUS accumulation and may ultimately suggest novel avenues for therapeutic investigation. Surprisingly, almost all of the genetic modifiers had no effect on TDP-43 toxicity in yeast. These key differences between FUS and TDP-43 will help guide the design of therapeutic interventions aimed at mitigating FUS aggregation in disease.

## Results

### FUS Forms Inclusions in the Yeast Cytoplasm and Is Toxic

To model aspects of FUS pathology in yeast, we first transformed yeast cells with a high-copy 2 micron (2 µ) plasmid containing human FUS fused to the yellow fluorescent protein (YFP; Figure 1B). Because TDP-43 was toxic to yeast [14], we placed FUS-YFP expression under the control of a tightly regulated galactose-inducible promoter (Figure 1B) to prevent deleterious effects during routine passage. After growing transformants in non-inducing conditions (raffinose media), we induced expression of FUS-YFP in galactose-containing media. Overexpression is a common tool to study the aggregation and toxicity of numerous proteins ranging from alpha-synuclein to TDP-43 [14],[39],[45]. It provides a method to elicit protein misfolding by increasing protein concentration and exceeding proteostatic buffers [46]. Moreover, overexpression is likely to yield key information because an established cause of several human neurodegenerative diseases is increased expression of aggregation-prone proteins, such as alpha-synuclein, amyloid precursor protein, and TDP-43 [47]–[49]. Following 4–6 h of induction, we visualized FUS-YFP localization by fluorescence microscopy (Figure 1C). Whereas the control, YFP alone, was localized diffusely throughout the cytoplasm and nucleus, FUS-YFP localized to the cytoplasm where it formed numerous foci (Figure 1C). FUS-YFP showed a similar cytoplasmic localization pattern when expressed from a low-copy galactose-inducible CEN plasmid (unpublished data). The FUS localization pattern was strikingly similar to that of TDP-43 in yeast (Figure 1C and [14]), in terms of size, shape, and quantity of foci in the cytoplasm (Figure 1C). Indeed when co-expressed in the same cell, FUS-YFP and TDP-43-CFP co-localized to the same cytoplasmic foci (Figure S2). Thus, TDP-43 and FUS inclusions partition to a similar compartment in yeast.

Next, we employed a weaker promoter (glyceraldehyde-3-phosphate dehydrogenase (GPD) promoter) to express FUS at lower levels. Here, FUS-YFP localized to both the nucleus and cytoplasm, where it was diffusely distributed (Figure S3). Similar results were seen with even weaker yeast promoters (CYC1 and NOP1; unpublished data). Thus, the FUS expression level plays a key role in FUS localization and aggregation in yeast. These data predict that sequence variants or copy number variants in the FUS gene that increase FUS expression might also contribute to ALS, FTLD-FUS, and other FUS proteinopathies. Indeed, a variant in the 3′UTR of the TDP-43 gene increases TDP-43 expression and contributes to FTLD-TDP [47]. Moreover, motor neurons express higher levels of FUS than other tissues, which might render them more vulnerable to FUS misfolding events [50].

In mammalian cells, FUS is normally restricted to the nucleus [51]–[55]. By contrast, in yeast, FUS is mostly localized to the cytoplasm. This difference suggests that the non-canonical FUS nuclear localization signal (NLS; amino acids 500–526) might not be very efficient in yeast. Indeed, in an accompanying manuscript, Ju et al. present data that directly support this hypothesis [56]. Alternatively, FUS might require post-translational modifications to localize to the nucleus, which do not occur in yeast. In an effort to restrict FUS to the nucleus, we fused a strong heterologous NLS (the SV40 NLS [57]) to the N-terminus of FUS. The SV40 NLS was sufficient to largely restrict FUS to the nucleus, but some cytoplasmic localization was also observed (Figure 1F). Importantly, restricting FUS to the nucleus eliminated aggregation (Figure 1F). Thus, FUS accumulation in the cytoplasm contributes to its aggregation. Despite the differences between FUS localization in yeast and mammalian cells, we can clearly use the genetically tractable yeast system to model FUS cytoplasmic aggregation, a critical pathological event in ALS and FTLD [17],[18]. Furthermore, defective nuclear import of FUS might be a key upstream event in ALS [52].

Having established that FUS, like TDP-43, forms cytoplasmic inclusions when expressed in yeast, we next asked if cytoplasmic aggregation of FUS was toxic. To assess FUS toxicity, we performed spotting assays on galactose media. Expressing FUS-YFP or untagged FUS inhibited growth, whereas YFP had no effect (Figure 1E). Thus, as for TDP-43, FUS expression in yeast was cytotoxic. Cytotoxicity correlated positively with cytoplasmic aggregation. First, expressing FUS at lower levels from the GPD promoter did not induce cytoplasmic FUS inclusions (Figure S3) and did not confer toxicity (unpublished data). Second, restricting FUS to the nucleus with the SV40 NLS (Figure 1F) greatly reduced toxicity (Figure 1G). These data suggest that cytoplasmic FUS aggregation is a critical pathological event in ALS and that neurodegeneration might be caused by a toxic gain of function in the cytoplasm.

Importantly, not every human RNA-binding protein aggregates and is toxic when expressed at high levels in yeast. Indeed, we expressed 132 human proteins containing RRMs in yeast. Of these, 35 (including TDP-43 and FUS) aggregated and were toxic (A.D.G. unpublished observations; Figure 1C). It will be important to determine whether any of these RRM-bearing proteins, aside from FUS and TDP-43, are connected to neurodegenerative disease. Moreover, it will be important to define whether common sequence determinants among these 35 RRM-bearing proteins promote aggregation and toxicity. One striking feature of FUS and TDP-43, as well as at least seven other human RNA-binding proteins that are toxic and aggregate in yeast, is the presence of a prion-like domain (Figure 1A; A.D.G. unpublished observations) [33].

### FUS Associates with Stress Granules and P-Bodies in Yeast

**Figure 2:**
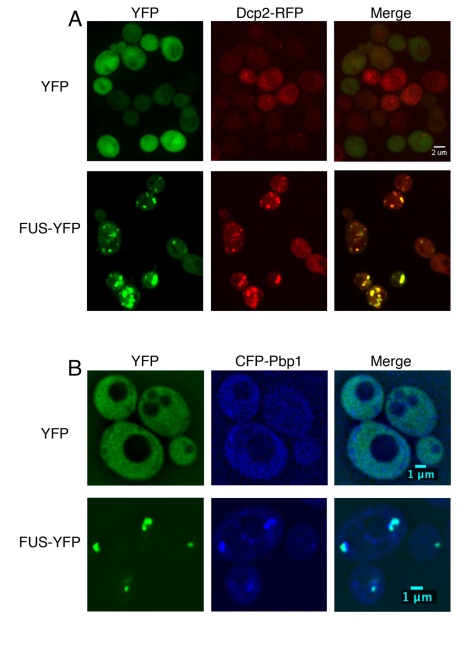
FUS associates with stress granules and P-bodies in yeast. (A) Yeast cells expressing YFP alone (top row) or FUS-YFP (bottom row). Dcp2-RFP was used to monitor P-body formation and localization. FUS-YFP expression induced the formation of P-bodies and FUS-YFP cytoplasmic localized to these structures. (B) FUS also induced the formation of and localized to stress granules, as monitored by a CFP-fusion to the stress granule protein Pbp1. Similar results were observed with independent P-body and stress granule markers, Lsm1 and Pub1, respectively (unpublished data).

We noticed that FUS-YFP cytoplasmic accumulations in yeast are highly dynamic under various growth conditions (Z.S., X.D.F., and A.D.G., unpublished observations). This dynamic behavior was reminiscent of RNA processing bodies (P-bodies) and stress granules. P-bodies and stress granules play important roles in regulating the translation, degradation, and localization of mRNAs. The pathways regulating the incorporation of RNAs and RNA-binding proteins into these structures are highly conserved from yeast to human [58]. Under various stress situations, including heat shock and oxidative stress, TDP-43 and FUS localize to these transient subcellular compartments and sites of RNA processing [59]–[62]. Moreover, even under normal conditions some ALS-linked FUS mutants localize to stress granules [51]–[55]. Thus, we tested whether FUS could induce stress granule or P-body formation in yeast and whether FUS localized to these structures. We expressed FUS-YFP or YFP alone in yeast cells harboring RFP- or CFP-tagged stress granule or P-body markers (Figure 2). To detect stress granules we used Pbp1-CFP and to detect P-bodies we used Dcp2-RFP [63]. Expressing YFP alone did not affect the localization of the P-body or stress granule components, which were diffuse under normal conditions (Figure 2A,B; unpublished data). However, FUS expression induced the formation of P-bodies and stress granules and FUS-YFP colocalized with both of these structures (Figure 2A,B). Thus, FUS localizes to and induces the formation of RNA granules in yeast as it does in human cells [51],[53]–[55]. These RNA granule assembly pathways are highly conserved from human to yeast. Thus, yeast provides a powerful system to dissect how FUS associates with these structures and to identify genetic and chemical modifiers of this process.

### Defining the Regions of FUS That Contribute to Aggregation and Toxicity in Yeast

**Figure 3:**
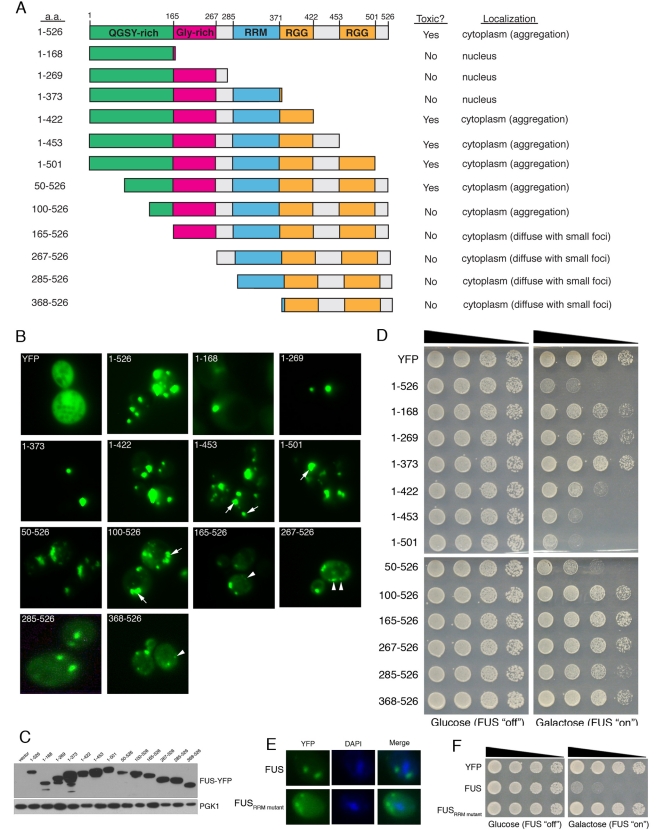
Defining the sequence features contributing to FUS aggregation and toxicity in yeast. (A) A diagram illustrating the domain structure of FUS along with truncation constructs used in this study. (B) Testing the effects of truncations on FUS localization by fluorescence microscopy. The C-terminal domain is required for cytoplasmic localization and aggregation (compare constructs 1–373 and 1–526). Arrows point to larger cytoplasmic FUS inclusions and arrowheads point to cells with more diffuse cytoplasmic FUS with small foci (see table in panel A). (C) Immunoblot showing expression levels of full-length FUS and each truncation. (D) The effects of truncations on toxicity were assessed by spotting assays. As for aggregation, the C-terminal region is required for toxicity but by itself is not sufficient (construct 368–526). The RRM, glycine-rich region and most of the prion-like domain (see [33]) are also required for FUS toxicity (compare constructs 1–501, 50–526, and 100–526). RRM, RNA recognition motif. (E) Mutating conserved phenylalanine residues in the FUS RRM to leucine to abolish RNA binding (FUS_RRM mutant_) does not affect FUS aggregation in yeast, however RNA binding is important for FUS toxicity because the FUS_RRM mutant_ eliminates toxicity in yeast (F).

To determine sequence features of FUS that were sufficient and necessary for aggregation and toxicity in yeast, we next performed a structure-function analysis. We recently used a similar approach for TDP-43 and determined that the C-terminal prion-like domain was required for aggregation and toxicity [14]. Underscoring the power of this approach, similar results have been reported for the C-terminal domain of TDP-43 in mammalian cells and in animal models [64],[65]. Moreover, all but one of the recently identified human ALS-linked TDP-43 mutations are located in this same C-terminal region [6]. We generated a series of FUS truncations (Figure 3A). We expressed each of the truncated FUS constructs as YFP-fusions and determined their subcellular localization (Figure 3B) and toxicity (Figure 3D). Immunoblotting confirmed that all of the fusion proteins were expressed at comparable levels (Figure 3C; unpublished data).

Full-length FUS formed multiple cytoplasmic inclusions in yeast (Figures 1C, 3B). Interestingly, removing the last 25 residues of FUS, which harbor most of the ALS-linked mutations [37], did not affect aggregation (Figure 3B, construct 1–501). This result is consistent with a recent report that a similar FUS truncation mutant (R495X) is connected with a severe ALS phenotype [51]. A larger C-terminal deletion also had little effect on cytoplasmic aggregation (Figure 3B, construct 1–453). Thus, C-terminal portions of FUS are not essential for cytoplasmic aggregation.

For TDP-43, the C-terminal prion-like domain is necessary but not sufficient for cytoplasmic aggregation [14]. TDP-43 also requires a portion of RRM2 (Figure 1A) [14]. However, for FUS, the N-terminal prion-like domain and the RRM resulted in an entirely nuclear localized protein (Figure 3B, construct 1–373; Figure S4). Adding back the first RGG domain (amino acids 371–422) was sufficient to restore cytoplasmic aggregation (Figure 3B, construct 1–422). Thus, in contrast to our findings with TDP-43 [14], the prion-like domain and the RRM of FUS (Figure 3B, construct 1–373) were insufficient to confer cytoplasmic aggregation. Additional C-terminal determinants within the first RGG domain are required to confer cytoplasmic aggregation (Figure 3B, construct 1–422).

Next, we asked if deletion of portions of the N-terminal prion-like domain of FUS, which spans the QGSY-rich domain and a portion of the Gly-rich domain (amino acids 1–239) (Figure 1A), prevented aggregation. Indeed, the generation of large cytoplasmic inclusions required most of the N-terminal QGSY-rich domain (Figure 3B, compare constructs 1–501, 50–526, 100–526, and 165–526) (Figure 3A,B). Deletion of the entire N-terminal QGSY-rich domain (construct 165–526) yielded mostly diffuse cytoplasmic staining with occasional small foci (Figure 3A,B). However, shorter N-terminal constructs comprising just the N-terminal QGSY-rich domain or this domain plus the Gly-rich domain did not aggregate and were localized in the nucleus (Figure 3B, constructs 1–168 and 1–269; Figure S4). Thus, the N-terminal prion-like domain of FUS is necessary but not sufficient for aggregation. Rather, FUS requires sequences in both the N-terminal region and the C-terminal region for robust formation of large cytoplasmic inclusions. Accordingly, large N-terminal deletions were diffusely localized within the cytoplasm, with only occasional small cytoplasmic puncta (Figure 3B, constructs 165–526, 267–526, 285–526, and 368–526). Thus, in distinction to TDP-43, which requires its C-terminal prion-like domain and a portion of RRM2 (Figure 1A) to aggregate in yeast [14], FUS requires its N-terminal prion-like domain, RRM, and first RGG domain to aggregate in yeast (Figures 1A, 3A). This key difference will have important implications for the design of therapeutic strategies aimed at preventing or reversing aggregation.

### The Domains of FUS Required for Aggregation in Yeast Contribute to Aggregation in Mammalian Cells

**Figure 4:**
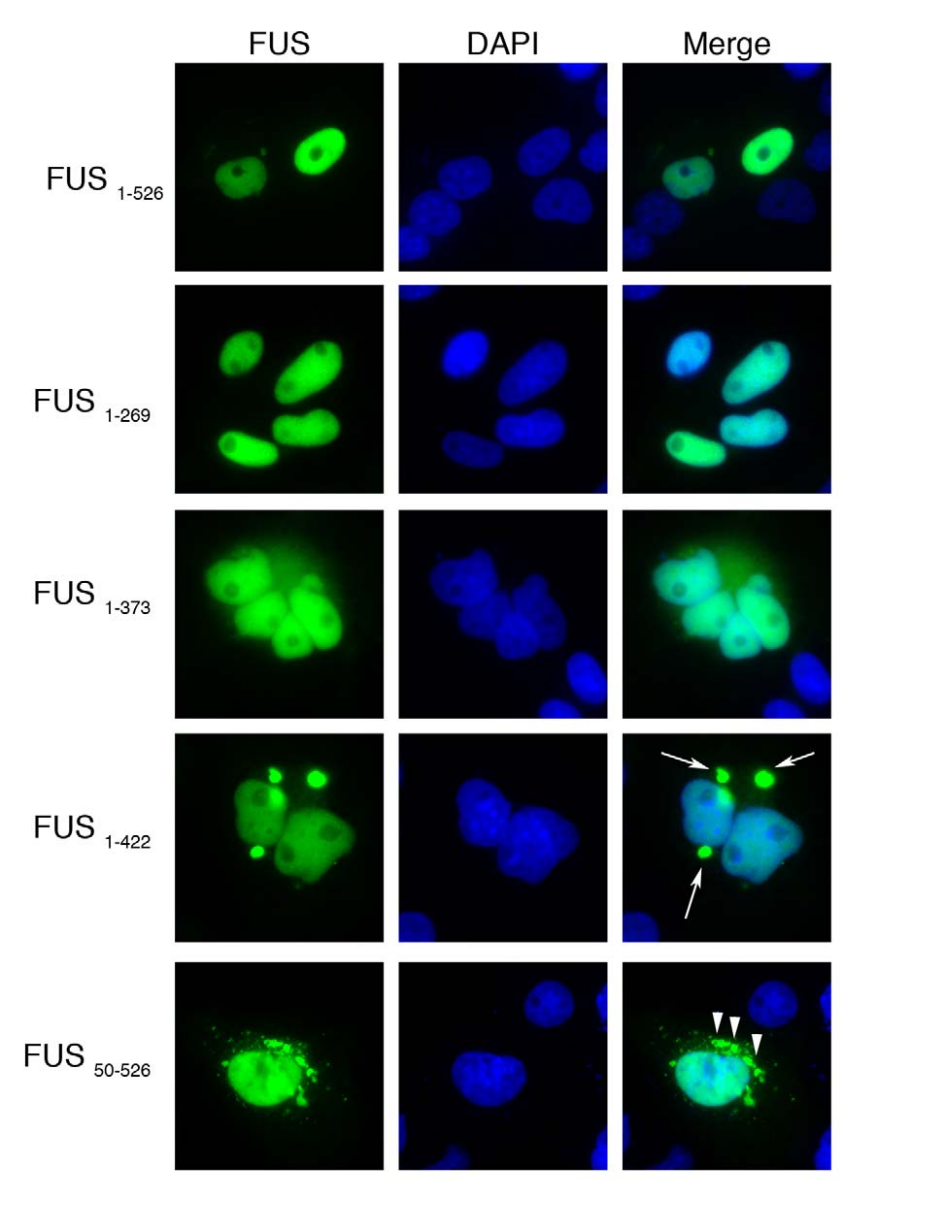
FUS domains that contribute to aggregation in mammalian cells. V5-tagged FUS expression constructs were transfected into COS-7 cells and their localization determined by fluorescence microscopy. Full-length FUS (1–526) localized to the nucleus, consistent with previous reports [17],[18],[51],[52]. Deletion constructs 1–269 and 1–373 also localized to the nucleus, consistent with our results in yeast (see Figure 2). Also as in yeast, the addition of sequences in the first FUS RGG domain resulted in FUS aggregation in the cytoplasm (construct 1–422, arrows). Construct 50–526 also aggregated in the cytoplasm, however the morphology of the inclusions (arrowheads) was distinct from that of 1–422.

Our domain mapping experiments in yeast indicate that the first RGG domain of FUS (amino acids 371–422) is important for driving aggregation (e.g., Figure 3B, compare constructs 1–373 and 1–422) and that sequences in the N-terminal prion-like domain (amino acids 1–239) are also important (e.g., Figure 3B, compare constructs 50–526 and 165–526). To test these predictions in mammalian cells, we transfected several of these deletion constructs (as C-terminal V5 epitope tag fusions) in COS-7 cells. In contrast to yeast cells, where full-length FUS (construct 1–526) forms cytoplasmic inclusions, and consistent with previous reports in mammalian cells [17],[18],[51],[52], full-length FUS localized almost exclusively to the nucleus, forming occasional cytoplasmic foci (Figure 4). This difference between the localization of full-length FUS in yeast (almost entirely cytoplasmic and forms inclusions) versus mammalian cells (almost entirely nuclear and diffuse) is also seen with TDP-43 (e.g., compare [66] and [14]) and might reflect differences in the efficacy of the FUS and TDP-43 nuclear localization signals in yeast and mammals. Indeed, Ju et al. demonstrate that the FUS NLS (amino acids 500–526) is ineffective in yeast [56].

Consistent with our yeast data, FUS constructs 1–269 and 1–373 localized almost exclusively to the nucleus in a diffuse pattern, although there was more cytoplasmic staining with 1–373 (Figure 4). These results were surprising since these constructs lack the C-terminal NLS defined in other studies [52],[54]. However, these results are consistent with those of Kino et al., who find that FUS 1–278 is localized to the nucleus and FUS 1–360 is localized to the nucleus as well as the cytoplasm [54]. These data suggest that additional determinants of nuclear localization exist in the FUS primary sequence. Indeed, scanning the FUS primary sequence using NLStradumus [67] revealed three NLS sequences in FUS comprising residues 241–251, 381–395, and 480–521. These two additional NLS sequences (241–251 and 381–395) might help explain why all of the FUS constructs in Figure 4 have some ability to localize to the nucleus.

Strikingly, as we observed in yeast, addition of the first RGG domain (construct 1–422) resulted in prominent cytoplasmic FUS aggregation in COS-7 cells (Figure 4). FUS construct 50–526 aggregated in yeast (Figure 3B) and mammalian cells (Figure 4). However, the morphology of the 50–526 inclusions was distinct from those formed by 1–422 (one or two large tight inclusions per cell with 1–422 versus numerous amorphous inclusions with 50–526). These data indicate that the domains of FUS required for aggregation in yeast (especially the first RGG domain) are also critical for FUS aggregation in mammalian cells. Moreover, these data validate the yeast system as a useful platform for interrogating mechanisms and genetic modifiers (see below) of FUS aggregation and toxicity.

### Defining the Domains of FUS Required for Toxicity in Yeast

Having determined the regions of FUS required for aggregation in yeast, we next determined which regions of FUS contributed to toxicity (Figure 3D). As with FUS aggregation, the last 25 amino acids of FUS, where many of the ALS-linked mutations occur [37], were not required for toxicity (Figure 3D, construct 1–501). Indeed, 1–501 was slightly more toxic than full-length FUS (Figure 3D). This finding is consistent with the severe ALS phenotype linked to FUS R495X [51]. Similar to TDP-43, the prion-like domain of FUS was required but not sufficient for toxicity (Figure 3D, compare constructs 1–526, 1–168, 1–269, and 267–526). As for aggregation, most of the N-terminal prion-like domain of FUS (amino acids 1–239) was needed for toxicity (Figure 3D, compare constructs 1–501, 50–526, and 100–526) and larger N-terminal deletions were not toxic (Figure 3D, compare constructs 165–526, 267–526, 285–526, and 368–526). However, unlike TDP-43 [14], adding back the RRM to the prion-like domain did not restore toxicity (Figure 3D, construct 1–373). Rather, for toxicity the RRM and the first RGG domain were required in addition to the prion-like domain (Figure 3D, compare constructs 1–373 and 1–422). However, 1–422 was not as toxic as full-length FUS, and additional C-terminal sequences were required to confer full toxicity (Figure 3D, compare constructs 1–422, 1–453, and 1–501). These findings are consistent with a pathogenic FUS truncation mutant (amino acids 1–466) connected with sporadic ALS [68].

Next, we tested whether FUS must bind RNA and aggregate to be toxic in yeast. Thus, we mutated conserved phenylalanine residues within the FUS RRM to leucine (Phe305, 341, 359, 368Leu) that would disrupt RNA binding [69]. These mutations were sufficient to mitigate toxicity but had no effect on cytoplasmic aggregation (Figure 3E,F). Analogous mutations to the RRMs of TDP-43 disable RNA binding [69] and also mitigate toxicity in yeast [16]. Taken together, these data indicate that the N-terminal prion-like domain, first RGG domain, and RRM (likely via RNA binding) of FUS contribute to toxicity. Identifying the specific RNA targets of FUS (for example, see [70]) will provide key insights into mechanisms of toxicity associated with FUS aggregation in disease. Overall, compared to TDP-43, FUS aggregation and toxicity in yeast is a more complex multi-domain process. Importantly, our studies define the prion-like FUS N-terminal domain and first RGG domain as potential targets to prevent or reverse FUS aggregation and toxicity.

### FUS Is Intrinsically Aggregation Prone

**Figure 5:**
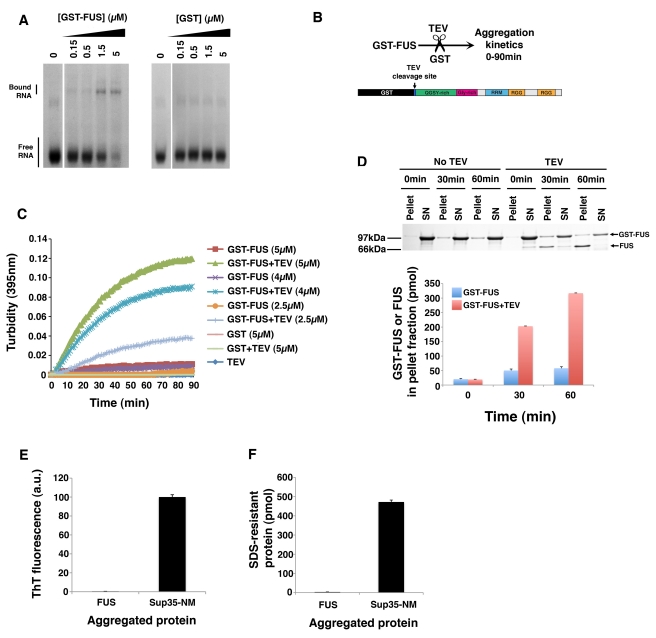
FUS is intrinsically aggregation prone. (A) RNA mobility shift experiments. ^32^P-labelled FUS RNA probe (see Materials and Methods) was incubated in the presence or absence of increasing amounts of GST-FUS or GST and resolved on a native gel to observe free and bound RNA species. (B) Schematic of FUS aggregation assay. TEV protease is added to remove the GST tag and untagged FUS aggregation kinetics are followed over 90 min. (C) GST-FUS or GST (2.5–5 µM) was incubated in the presence or absence of TEV protease at 22°C for 0–90 min. Turbidity measurements were taken every minute to assess the extent of aggregation. Values represent means (*n* = 3). (D) GST-FUS (5 µM) was incubated in the presence or absence of TEV protease at 22°C for 0–60 min. At the indicated times, reactions were processed for sedimentation analysis. Pellet and supernatant fractions were resolved by SDS-PAGE and stained with Coomassie Brilliant Blue. A representative gel is shown. Note that cleaved FUS partitions mostly to the pellet fraction, whereas GST-FUS remains in the supernatant (SN) fraction. The amount of GST-FUS or FUS in the pellet fraction was determined by densitometry in comparison to known quantities of GST-FUS or FUS. Values represent means ± SEM (*n* = 4). (E) FUS (5 µM) was aggregated as in (C) for 60 min and processed for Thioflavin-T (ThT) fluorescence and compared to the ThT fluorescence of assembled Sup35-NM fibers (5 µM monomer). Values represent means ± SEM (*n* = 3). (F) FUS (5 µM) was aggregated as in (C) for 60 min. The amount of SDS-resistant FUS was then determined and compared to the amount of SDS-resistant Sup35-NM in assembled Sup35-NM fibers (5 µM monomer). Values represent means ± SEM (*n* = 3).

To determine whether FUS is intrinsically prone to aggregation, we purified bacterially expressed recombinant FUS as a soluble protein under native conditions. However, expression of various constructs including N- and C-terminal His-tagged FUS in various bacterial strains failed to yield soluble protein. The solubility of various proteins, including TDP-43 and polyglutamine, can be enhanced by the addition of a glutathione-S-transferase (GST) tag [15],[41],[71]. Even so, FUS bearing a C-terminal GST-tag was also insoluble in various bacterial strains. Fortunately, an N-terminal GST-tag allowed FUS to be purified as a soluble protein under native conditions. GST-FUS remained soluble for extended periods and was competent to bind RNA in mobility shift assays (Figure 5A). To study FUS aggregation, we added tobacco etch virus (TEV) protease to cleave at a single unique site and specifically remove the N-terminal GST-tag (Figure 5B). This strategy has been utilized successfully to study the aggregation of extremely aggregation-prone proteins, including polyglutamine [41],[43]. Upon addition of TEV protease, FUS aggregated extremely rapidly (Figure 5C). By contrast, GST-FUS remained predominantly soluble (Figure 5C). Under identical conditions neither GST nor TEV protease aggregated (Figure 5C). Aggregation was dependent on FUS concentration in three ways: at higher FUS concentrations, the maximum amplitude or endpoint of turbidity was increased, the length of lag phase was reduced and the rate of aggregation during assembly phase was accelerated (Figure 5C). Sedimentation analysis revealed that after addition of TEV protease, FUS entered the pellet fraction, whereas GST-FUS remained largely soluble (Figure 5D). Indeed, there was very little FUS in the supernatant fraction at any time, indicating that aggregation occurred rapidly after proteolytic liberation of FUS from GST (Figure 5D). The aggregates formed by FUS did not react with the amyloid-diagnostic dye Thioflavin-T and were SDS-soluble, in contrast to those formed by NM, the prion domain of yeast prion protein Sup35 (Figure 5E,F). Thus, pure FUS forms aggregates that are likely non-amyloid in nature, just like the aggregated species of FUS observed in ALS and FTLD patients [5],[72],[73].

**Figure 6:**
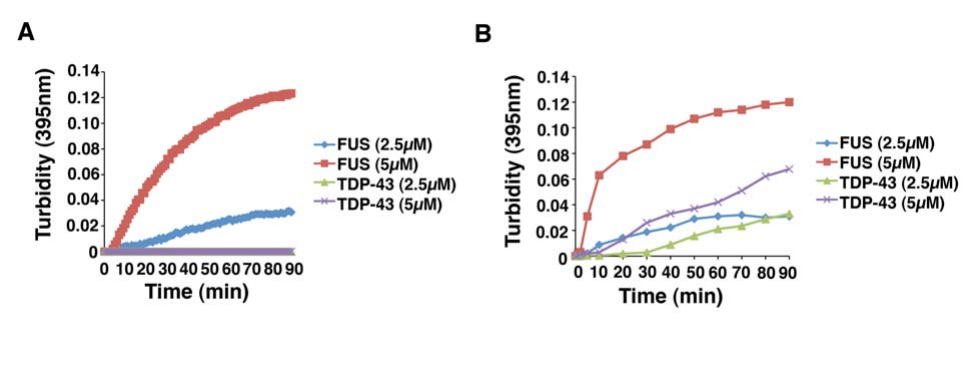
FUS aggregates more rapidly than TDP-43 in vitro. (A) GST-FUS or GST-TDP-43 (2.5 or 5 µM) was incubated in the presence of TEV protease at 22°C for 0–90 min. Turbidity measurements were taken every minute to assess the extent of aggregation. A dataset representative of three replicates is shown. (B) GST-FUS or GST-TDP-43 (2.5 or 5 µM) was incubated in the presence of TEV protease at 22°C with agitation (700 rpm) for 0–90 min. The extent of aggregation was determined by turbidity. A dataset representative of three replicates is shown.

The rapid aggregation of FUS occurred without agitation of the reaction (Figures 5C, 6A). Remarkably, under these conditions, even TDP-43 did not aggregate (Figure 6A). TDP-43 requires many hours to aggregate unless the reaction is agitated [15]. Agitation had little effect on the rate of FUS aggregation (Figure 6A,B), indicating that under these conditions FUS aggregation is energetically favorable. Even when the reaction was agitated, TDP-43 aggregation was still considerably slower than FUS aggregation (Figure 6B). In particular, the lag period prior to aggregation was longer for TDP-43 than for FUS (Figure 6B). This extended lag period was not due to different rates of FUS or TDP-43 cleavage by TEV protease, which were extremely similar (unpublished data). Rather, nucleation of aggregation is apparently more rate limiting for TDP-43 than it is for FUS. Collectively, these data suggest that, even in comparison to TDP-43, FUS is extremely aggregation prone. These data are also in keeping with the higher prion-like domain score of FUS compared to TDP-43 [33]. In vivo, such rapid FUS aggregation is most likely precluded by the proteostasis network [46]. However, FUS likely escapes these safeguards in disease situations where proteostatic buffers may have declined with age or because of environmental triggers. Irrespective of the factors that may elicit FUS aggregation in disease, pure protein assays akin to the one we report here have been powerful tools to dissect the mechanisms underlying the aggregation of various disease-connected proteins, including TDP-43 and polyglutamine [15],[41],[43].

### The Prion-Like Domain and First RGG Domain of FUS Are Important for Aggregation

**Figure 7:**
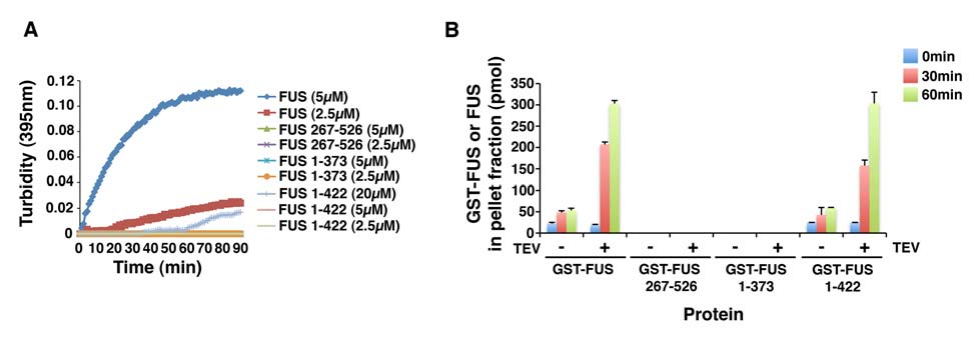
Defining the domain requirements for the aggregation of pure FUS. (A) GST-FUS, GST-FUS 267–526, GST-FUS 1–373 (2.5 µM or 5 µM), or GST-FUS 1–422 (2.5 µM, 5 µM, or 20 µM) were incubated in the presence of TEV protease at 22°C for 0–90 min. Turbidity measurements were taken every minute to assess the extent of aggregation. A dataset representative of three replicates is shown. (B) GST-FUS, GST-FUS 267–526, GST-FUS 1–373, or GST-FUS 1–422 (5 µM) were incubated in the presence or absence of TEV protease at 22°C for 0–60 min. At the indicated times, reactions were processed for sedimentation analysis. Pellet and supernatant fractions were resolved by SDS-PAGE and stained with Coomassie Brilliant Blue. The amount of GST-FUS or FUS in the pellet fraction was determined by densitometry in comparison to known quantities of GST-FUS or FUS. Values represent means ± SEM (*n* = 3).

Next, we determined how the N- and C-terminal domains of FUS contribute to aggregation of the pure protein. Consistent with observations in yeast (Figure 3B), deletion of the N-terminal prion-like domain of FUS yielded protein (267–526) that remained soluble over the time course of the assay as determined by turbidity and sedimentation analysis (Figure 7A,B). These data suggest that the prion-like domain of FUS is required for aggregation. Curiously, however, but also consistent with observations in yeast, a protein bearing the prion-like domain and adjacent C-terminal sequences (1–373) did not aggregate under these conditions (Figure 7A,B). Even at higher concentrations (20 µM), neither FUS 267–526 nor FUS 1–373 aggregated. Moreover, if the reaction was subsequently agitated at 700 rpm for an additional 60 min neither FUS 267–526 nor FUS 1–373 aggregated.

Next, we tested FUS 1–422, a minimal fragment of FUS able to confer toxicity *and* aggregation in yeast (Figure 3B,D). FUS 1–422 aggregated with similar kinetics to full-length FUS as determined by sedimentation analysis (Figure 7B). Curiously, however, at these concentrations (2.5–5 µM) FUS 1–422 aggregates did yield a signal by turbidity (Figure 7A). Higher concentrations of FUS 1–422 (20 µM) were required to generate aggregates detectable by turbidity (Figure 7A). These concentration differences in the turbidity measurements for full-length FUS and FUS 1–422 suggest that there are large disparities in the sizes of the aggregates formed by these two proteins because turbidity readily detects large but not small aggregates [74]–[76]. A similar finding has been made with PrP, where deletion of the N-terminal domain reduces the formation of larger turbid aggregates, without affecting the formation of smaller aggregates [74]. These data suggest that the C-terminal region, comprising amino acids 423–526, while dispensable for aggregation per se (Figure 7B), promotes the formation of large macroscopic aggregates of FUS that are detected by turbidity (Figure 7A).

### Pure FUS Aggregates Resemble FUS Aggregates in Degenerating Neurons of ALS Patients

**Figure 8:**
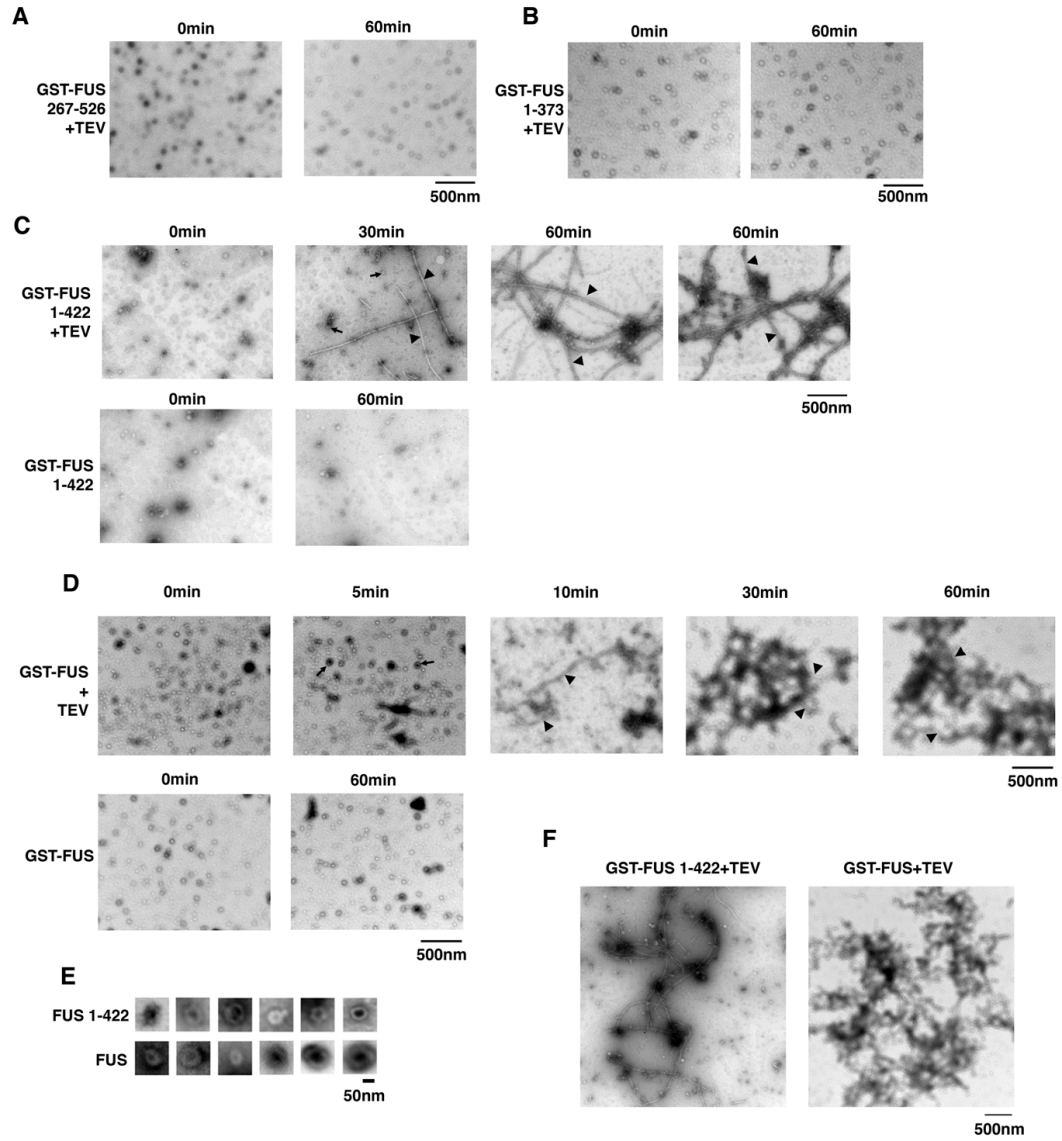
Pure FUS aggregates resemble FUS aggregates in degenerating motor neurons of ALS patients. (A, B) GST-FUS 267–526 (2.5 µM) (A) or GST-FUS 1–373 (2.5 µM) (B) were incubated in the presence of TEV protease at 22°C for 0 or 60 min and processed for EM. Bar, 500 nm. (C) GST-FUS 1–422 (2.5 µM) was incubated in the absence or presence of TEV protease at 22°C for 0–60 min. At the indicated times, reactions were processed for EM. In the absence of TEV protease, very little aggregation occurs. In the presence of TEV protease, pore-shaped oligomers (arrows) and filamentous polymers (arrowheads) rapidly assemble. At 60 min, the filamentous structures stay well separated but are sometimes associated with smaller FUS 1–422 oligomers. Bar, 500 nm. (D) GST-FUS (2.5 µM) was incubated in the absence or presence of TEV protease at 22°C for 0–60 min. At the indicated times, reactions were processed for EM. In the absence of TEV protease, very little aggregation occurs. In the presence of TEV protease, pore-shaped oligomers (arrows) and filamentous polymers (arrowheads) rapidly assemble. The filamentous structures often form higher order network structures by 30 and 60 min. (E) Gallery of pore-shaped FUS 1–422 oligomers formed after 30 min and pore-shaped FUS oligomers formed after 10 min. Bar, 50 nm. (F) Lower magnification view of filamentous FUS 1–422 and FUS aggregates formed after 60 min in the presence of TEV protease. Note that FUS aggregates accumulate as larger networks that conglomerate into large aggregates, whereas FUS 1–422 filaments remain well separated. This difference in morphology likely explains why FUS aggregates generate a larger turbidity signal than FUS 1–422 aggregates. Bar, 500 nm.

Electron microscopy (EM) confirmed that pure FUS 1–373 and FUS 267–526 do not form aggregated species in isolation (Figure 8A,B). Rather, these proteins persist as small oligomeric particles (Figure 8A,B). In the absence of TEV protease, both FUS and FUS 1–422 did not aggregate but remained as small oligomeric species (Figure 8C,D). After addition of TEV protease, FUS and FUS 1–422 rapidly populated oligomeric forms, which adopted a pore-like conformation reminiscent of pathological oligomers formed by TDP-43, α-synuclein, and Aβ42 (Figure 8E) [15],[42]. FUS 1–422 rapidly aggregated in an ordered manner to generate separated filamentous structures (Figure 8C). Likewise, full-length FUS also rapidly formed linear polymers (Figure 8D). In both cases, these filaments were approximately 15–20 nm in diameter and could extend several micrometers in length (Figure 8C,D). Consistent with turbidity measurements, the polymers formed by full-length FUS became tangled and stacked against one another to form extremely large and complex macroscopic networks (Figure 8D,F). FUS 1–422 polymers remained more separated with limited lateral interaction (Figure 8C,F). These ultrastructural observations explain why FUS 1–422 aggregates are more difficult to detect by turbidity.

Importantly, the filamentous structures formed by both FUS and FUS 1–422 bear striking resemblance to the FUS aggregates observed in the degenerating motor neurons of ALS patients [21],[77]. In motor neurons of patients with juvenile ALS, FUS forms filamentous aggregates with a uniform diameter of 15–20 nm, which are often associated with small granules [21],[77]. The filamentous structures formed by FUS and FUS 1–422 in isolation (Figure 8C,D,F) are extremely similar to those observed in spinal motor neurons in Figure 3C of Huang et al. [21]. In vitro, small FUS or FUS 1–422 oligomers are often found clustered up against the filamentous structures (Figure 8C,D,F). These oligomers may correspond to the granular structures observed in association with filamentous FUS aggregates in motor neurons of ALS patients [21],[77]. In sum, these observations suggest that in isolation FUS is intrinsically capable of forming the aggregated structures observed in motor neurons of ALS patients.

Taken together, the biochemical and EM data suggest that FUS aggregation requires multiple domains in both N- and C-terminal regions. Specifically, determinants in the N-terminal prion-like domain (1–239) and the first C-terminal RGG domain (374–422) are essential for the formation of filamentous structures. More C-terminal regions (423–526) are then required for the formation of large macroscopic aggregates detected by turbidity.

### ALS-Linked FUS Mutations Do Not Affect Aggregation or Toxicity

**Figure 9:**
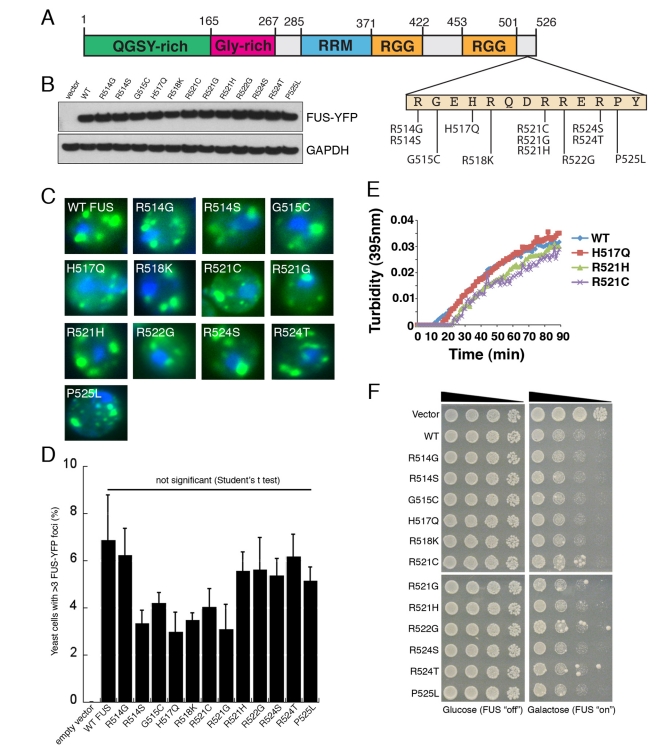
The effect of ALS-linked FUS mutations on aggregation and toxicity. (A) Diagram indicating disease-associated FUS mutations tested in this study. (B) Immunoblot showing equivalent expression levels of WT or mutant FUS. Glyceraldehyde-3-phosphate dehydrogenase (GAPDH) was used as a loading control. (C) ALS-linked mutations did not significantly affect FUS aggregation in yeast. (D) The effect of ALS-linked FUS mutations on aggregation in yeast cells was quantified by counting the number of cells containing >3 FUS-YFP foci (as in [15] for TDP-43). Values represent means ± SEM (*n*≥3, at least 200 cells per sample). As for FUS toxicity, these ALS-linked mutations did not significantly enhance FUS aggregation, in contrast to TDP-43 mutations, which did increase aggregation [15]. (E) GST-FUS, GST-FUS H517Q, GST-FUS R521H, or GST-FUS R521C (2.5 µM) was incubated in the presence of TEV protease at 22°C for 0–90 min. Turbidity measurements were taken every minute to assess the extent of aggregation. A dataset representative of three replicates is shown. (F) Spotting assay to compare the toxicity of WT and mutant FUS. Serial dilutions of yeast cells transformed with galactose-inducible empty vector, WT, or mutant FUS-YFP constructs. Transformants were spotted on glucose (non-inducing) or galactose (inducing) containing agar plates, and growth was assessed after 3 d. In contrast to TDP-43 [15], the ALS-linked FUS mutations did not enhance FUS toxicity in yeast.

FUS mutations have been connected with some familial and sporadic ALS cases [37]. We next used the yeast model to test the effects of some of these mutations on FUS aggregation and toxicity (Figure 9A). For TDP-43, we have used this approach to determine that ALS-linked mutations increase TDP-43 aggregation and toxicity [15]. This increased toxicity of mutant TDP-43 in yeast has been supported by independent studies in mammalian cells and animal models [9],[78]–[80]. To assess aggregation, we expressed YFP-tagged fusions of WT FUS and 12 different ALS-linked FUS mutants in yeast. These FUS variants were all expressed at similar levels (Figure 9B). Moreover, comparison of the number of proportion of yeast cells with three or more foci revealed that ALS-linked FUS mutations do not promote FUS aggregation in yeast (Figure 9C,D). Indeed, FUS aggregation was slightly reduced in various ALS-linked FUS variants, although this reduction was not statistically significant (Figure 9C,D). Consistent with these observations, the ALS-linked FUS variants—H517Q, R521C, and R521G—aggregated with very similar kinetics to WT in pure protein aggregation assays, although aggregation was slightly retarded in these mutants (Figure 9E). Collectively, these data suggest that this set of ALS-linked FUS mutations, clustered in the extreme C-terminal region of FUS, do not promote FUS aggregation per se. Furthermore, we did not observe any significant difference in toxicity between WT and ALS-linked FUS variants (Figure 9F). These data are in contrast to TDP-43, where several ALS-linked mutations promote aggregation and toxicity [15].

It seems likely that in disease, these C-terminal ALS-linked FUS mutations promote pathological events that are primarily upstream of aggregation and toxicity. One obvious upstream event is mislocalization to the cytoplasm. Indeed, studies in mammalian cells suggest that ALS-linked FUS mutations can disrupt nuclear import [52]. In yeast, FUS is already localized predominantly to the cytoplasm (Figures 1C, 9C), so in this setting the ALS-linked mutants are no more toxic than WT (Figure 9C,D,F). Thus, even though FUS and TDP-43 are related RNA-binding proteins, the mechanisms by which ALS-linked mutations contribute to disease might be different for each protein [52]. Consequently, different therapeutic strategies might be needed for FUS and TDP-43 proteinopathies. To examine this idea further, we performed two genome-wide screens in yeast to (1) identify genetic modifiers of FUS toxicity and (2) determine whether genetic modifiers of FUS toxicity also affected TDP-43 toxicity.

### A Yeast Genome-Wide Overexpression Screen Identifies Modifiers of FUS Toxicity

Of the many experimental benefits afforded by the yeast system [13], the chief advantage is the ability to perform high-throughput genetic modifier screens. Therefore, to provide insight into cellular mechanisms underpinning FUS toxicity, we performed two unbiased yeast genetic modifier screens to identify genes that enhance or suppress FUS toxicity. We reasoned that the genes identified by these screens would illuminate cellular pathways perturbed by abnormal FUS accumulation and suggest potential novel targets for therapeutic intervention. Similar approaches have elucidated modifiers of the Parkinson's disease protein α-synuclein [39],[40],[45],[81], a mutant form of the Huntington's disease protein huntingtin [44],[45], and more recently, the ALS protein TDP-43 ([16]; A. Elden and A.D.G. unpublished). In the latter example, the yeast system allowed definition of a common genetic risk factor for ALS in humans [16].

**Figure 10:**
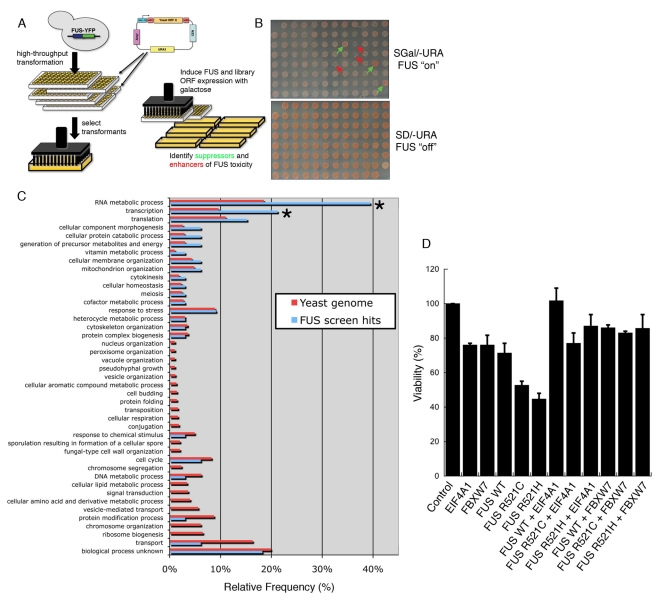
Yeast plasmid overexpression screen identifies suppressors and enhancers of FUS toxicity. (A) Schematic of yeast genetic screen. Yeast cells harboring an integrated galactose-inducible FUS-YFP cassette were individually transformed with a library of 5,500 yeast open reading frames (ORFs) and spotted onto galactose plates to induce expression of FUS and each gene from the library. (B) A representative plate from the yeast screen. Each spot represents a yeast strain expressing FUS along with one gene from the library. Examples of genes that suppressed FUS toxicity (improved growth) are indicated by green arrows and enhancers of toxicity (inhibited growth) are indicated by red arrows. (C) A histogram indicating the functional categories of genes enriched as hits in the screen compared to the yeast genome. Genes involved in transcription and RNA metabolism were significantly overrepresented as hits in the screen (indicated by *). (D) Human homologs of two FUS toxicity modifier genes from the yeast screen, FBXW7 and EIF4A1, suppressed FUS toxicity in human cells (HEK293T), when co-transfected with FUS or ALS-linked FUS mutants, R521C and R521H. Cell viability was assessed by MTT assay. Values represent means ± S.D. (*n* = 3).

First, we performed a plasmid overexpression screen (Figure 10A). We individually transformed 5,500 yeast genes, which comprise the Yeast FLEXGene plasmid overexpresssion library [82], into a yeast strain harboring an integrated galactose-inducible FUS expression plasmid. We then identified yeast genes that suppressed or enhanced FUS toxicity when overexpressed (Figure 10B). We repeated the screen three independent times and only selected hits that reproduced all three times. Genes from the screen that enhanced FUS toxicity, but also caused toxicity when overexpressed in WT yeast cells, were eliminated because these were unlikely to be specific to FUS. We also eliminated certain genes involved in carbohydrate metabolism or galactose-regulated gene expression because, based on previous screens with this library, we have found that they simply affect expression from the galactose-regulated promoter and are unlikely to relate to FUS biology. Indeed, most of these were also recovered as hits in screens with a galactose-regulated toxic huntingtin, α-syn or TDP-43 ([16],[39],[44],[45],[81]; A. Elden and A.D.G. unpublished). Finally, we retested 10 random plasmids (six suppressors and four enhancers) by transforming them into a fresh yeast strain harboring the integrated FUS expression plasmid and performed spotting assays and all 10 of these were confirmed (Figure S5).

Following the above validation and filtering procedures, we identified 24 genes that suppressed and 10 genes that enhanced FUS toxicity when overexpressed (Table 1). The largest functional class enriched in the screen included RNA-binding proteins and proteins involved in RNA metabolism (Figure 10C). Thus, RNA metabolic pathways play a key role in FUS pathogenesis. Importantly, of 71 genes from this library that modify α-synuclein toxicity in yeast [39],[40], only two (Cdc4 and Tps3) affected FUS toxicity. This lack of overlap underscores the specificity of the screen for FUS biology and pathobiology. Moreover, this specificity indicates that the screen does not simply identify generic cellular responses to misfolded proteins. Even more remarkably, out of the 40 yeast genes that we have found to modify TDP-43 toxicity when overexpressed ([16] and A. Elden and A.D.G. unpublished observations), only two (Fmp48 and Tis11) affected FUS toxicity. Thus, despite being similar RNA-binding proteins, the mechanisms by which FUS and TDP-43 contribute to disease are likely to be very different.

**Table 1:** Yeast genes that suppress or enhance FUS toxicity when overexpressed.

Effect	Gene	Human Homolog	Function
Suppressor	CDC4	FBXW7	F-box protein required for G1/S and G2/M transition
Suppressor	CUE2		Protein of unknown function
Suppressor	ECM32	UPF1	DNA dependent ATPase/DNA helicase, involved in modulating translation termination
Suppressor	EDC3		Non-essential conserved protein of unknown function, plays a role in mRNA decapping
Suppressor	FHL1		Putative transcriptional regulator, required for rRNA processing
Suppressor	FMP48	STK36	Mitochondrial protein of unknown function
Suppressor	NAM8	TRNAU1AP	RNA binding protein, component of the U1 snRNP protein
Suppressor	PAB1	PABPC4	Poly(A) binding protein, part of the 3′-end RNA-processing complex, involved in stress granule formation
Suppressor	PIG1		Putative targeting subunit for the type-1 protein phosphatase Glc7p
Suppressor	SBP1		Nucleolar single-strand nucleic acid binding protein, associates with small nuclear RNAs
Suppressor	SEY1		Protein of unknown function, contains two predicted GTP-binding motifs
Suppressor	SKO1		Basic leucine zipper (bZIP) transcription factor of the ATF/CREB family
Suppressor	SYN8	STX8	Endosomal SNARE related to mammalian syntaxin 8
Suppressor	TIF2	EIF4A1	Translation initiation factor eIF4A, RNA helicase that couples ATPase activity to RNA binding and unwinding, involved in stress granule formation
Suppressor	TIF3	EIF4B	Translation initiation factor eIF-4B, has RNA annealing activity, contains an RNA recognition motif and binds to single-stranded RNA, involved in stress granule formation
Suppressor	TIS11		mRNA-binding protein involved in iron homeostasis
Suppressor	TPS3		Regulatory subunit of trehalose-6-phosphate synthase/phosphatase complex
Suppressor	TRM11	TRMT11	Catalytic subunit of an adoMet-dependent tRNA methyltransferase complex
Suppressor	VHR1		Transcriptional activator
Suppressor	YHR151C		Unknown
Suppressor	YOR062C		Unknown
Suppressor	YPR147C	C2orf43	Unknown
Suppressor	ZDS2		Protein that interacts with silencing proteins at the telomere, involved in transcriptional silencing
Enhancer	CLB2	CCNB1	B-type cyclin involved in cell cycle progression
Enhancer	CST6		Basic leucine zipper (bZIP) transcription factor of the ATF/CREB family
Enhancer	FZO1		Mitochondrial integral membrane protein involved in mitochondrial fusion and maintenance of the mitochondrial genome
Enhancer	HOF1		Bud neck-localized protein required for cytokinesis
Enhancer	INM1	IMPA1	Inositol monophosphatase
Enhancer	IRC3	EIF4A3	Putative RNA helicase of the DEAH/D-box family
Enhancer	NAB3		Single stranded RNA binding protein, required for termination of non-poly(A) transcripts and efficient splicing
Enhancer	PET111		Specific translational activator for the COX2 mRNA, located in the mitochondrial inner membrane
Enhancer	TRM5	TRMT5	tRNA methyltransferase
Enhancer	YMR166C	SLC25A26	Predicted transporter of the mitochondrial inner membrane

Several of the yeast genes that modified FUS toxicity have human homologs. Thus, pathways involved in FUS toxicity in yeast are likely conserved to man. Interestingly, FUS has recently been shown to co-localize with stress granules in transfected cells [51],[52]. Furthermore, cytoplasmic FUS-positive inclusions in ALS and FTLD-U patients contain stress granule markers [51],[52]. Stress granules and P-bodies are transient cytoplasmic structures containing RNAs and RNA binding proteins, including translation initiation factors and the polyA-binding protein (PABP-1), which are sites where cells sequester mRNAs, during situations of stress, to inhibit translation initiation [83]. Notably, we identified two translation initiation factors (Tif2 and Tif3) and Pab1, the yeast homolog of human PABP-1, which is involved in stress granule assembly in yeast, as suppressors of FUS toxicity (Table 1). Thus, in addition to being markers of FUS-positive inclusions in disease, stress granule components might play an important role in mediating FUS toxicity. Approaches aimed at manipulating stress granule assembly might be an effective therapeutic approach.

### Overexpression Suppressors Isolated from Yeast Also Suppress FUS Toxicity in Mammalian Cells

As an initial step to extend our findings from yeast to mammalian cells, we selected genes from our overexpression screen for further analysis in a mammalian cell culture FUS toxicity model. We tested two distinct suppressor genes, FBXW7 and EIF4A1, which are the human homologs of yeast Cdc4 and Tif2, respectively (Table 1). We transfected HEK293T cells with WT FUS or two ALS-linked FUS mutants, R521C and R521H. The FUS mutants were more toxic than WT FUS, which only slightly reduced viability (Figure 10D). Co-transfection with FBXW7 or EIF4A1 suppressed toxicity of WT FUS as well as the ALS-linked FUS mutants (Figure 10D). Similar results were observed in COS-7 cells (unpublished data). The FUS toxicity modifier genes and pathways identified in our yeast screens will have to be validated in neuronal cells and eventually animal models. However, the ability of FBXW7 and EIF4A1 to suppress toxicity in human cells, which are separated from yeast by ∼1 billion years of evolution, provides evidence that highly conserved genetic interactions involving FUS, discovered in yeast, can be highly relevant to mammalian cells.

### A Yeast Genome-Wide Deletion Screen Identifies Modifiers of FUS Toxicity

**Figure 11:**
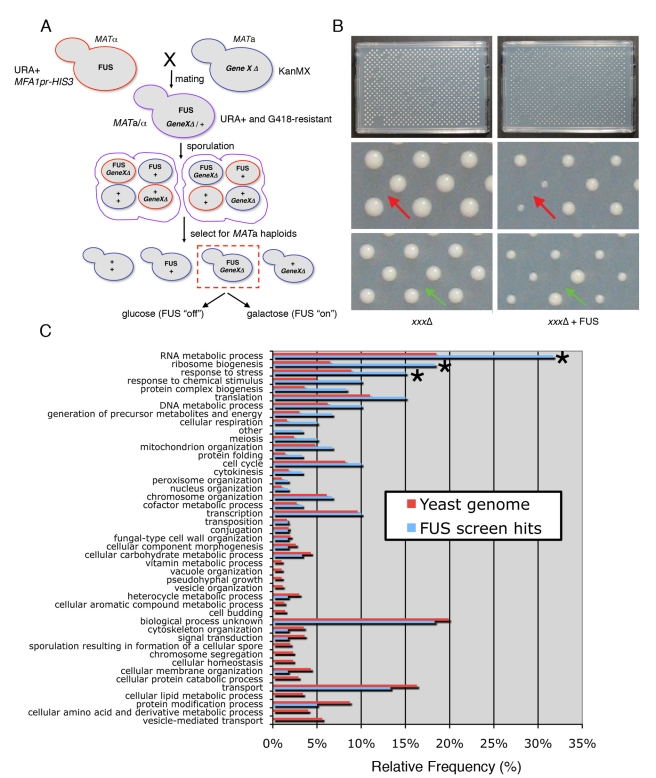
Yeast deletion screen identifies suppressors and enhancers of FUS toxicity. (A) Schematic of yeast deletion screen, based on [87]. The galactose-inducible FUS expression construct (pAG416Gal-FUS-YFP) was introduced into MATα strain Y7092 to generate the query strain. This query strain was mated to the yeast haploid deletion collection of non-essential genes (MATa, each gene deleted with KanMX cassette (confers resistance to G418)). Mating, sporulation, and mutant selection were performed using a Singer RoToR HDA (Singer Instruments, Somerset, UK). Haploid mutants harboring the FUS expression plasmid were grown in the presence of glucose (FUS expression “off”) or galactose (FUS expression “on”). Following growth at 30°C for 2 d, plates were photographed and colony sizes measured by ImageJ image analysis software, based on [104]. (B) A representative plate from the deletion screen. Left is glucose (deletion alone, e.g. xxxΔ) and right is galactose (deletion + FUS expression, e.g. xxxΔ + FUS). Each plate contains 384 different strains pinned in duplicate (768 total). The red arrows point to an aggravating genetic interaction (toxicity enhancer), in which the gene deletion + FUS grows slower than FUS or the deletion alone. The green arrows point to an alleviating genetic interaction (toxicity suppressor), in which the gene deletion + FUS grows better than FUS or the deletion alone. (C) A histogram indicating the functional categories of genes enriched as hits in the screen compared to the yeast genome. Genes involved in RNA metabolism, ribosome biogenesis, and cellular stress responses were significantly overrepresented as hits in the screen (indicated by *).

To complement the yeast overexpression screen, we also performed a deletion screen. The yeast genome contains ∼6,000 yeast genes and ∼4,850 of these are non-essential [84],[85]. We used synthetic genetic array (SGA) analysis [86],[87] to introduce a FUS expression plasmid into each non-essential yeast deletion strain by mating (Figure 11A). Following sporulation, we selectively germinated meiotic progeny containing both the FUS plasmid and the gene deletion. We compared growth of each strain on glucose (FUS expression “off”) to that on galactose (FUS expression “on”). We identified some yeast deletions that enhanced FUS toxicity (aggravating interaction) and others that suppressed toxicity (alleviating interaction) (Figure 11B). As for the overexpression screen, we repeated the deletion screen three independent times and only selected hits that reproduced all three times and filtered out deletion strains that grew poorly on galactose-containing media, even in the absence of FUS (using published data on yeast deletion strain fitness on galactose and in house measurements of the yeast deletion collection grown on galactose). Genetic interactions were further confirmed by random spore analysis and the integrity of the deletions verified by sequencing the deletion specific bar codes. We also independently confirmed six random hits by remaking the deletions, confirming the deletions by PCR, and then transforming those deletion strains with the FUS expression plasmid and performing spotting assays.

We indentified 36 deletions that suppressed FUS toxicity and 24 that enhanced toxicity (Table 2). Deletions of yeast genes involved in RNA metabolic processes, ribosome biogenesis, and cellular stress responses were enriched as hits (Figure 11C). Many of these genes have human homologs (Table 2). One interesting deletion suppressor was Sse1, a member of the Hsp70 chaperone family, which promotes Sup35 prion formation [88] and might also promote FUS aggregation. Two other notable deletion suppressors were Pub1 (TIAL1 in human) and Lsm7 (LSM7 in human), components of stress granules and P-bodies, respectively. Furthermore, TIAL1 (and Pub1) contains a prion-like domain [32],[89], which can template the aggregation of the polyQ protein huntingtin [90], suggesting that FUS aggregation and cytoplasmic sequestration might be templated by similar mechanisms [24],[91]. Again, as for the plasmid overexpression screen, genetic manipulations that affect stress granule components are sufficient to mitigate FUS toxicity. And, as for the overexpression screen, there was little overlap between the FUS and TDP-43 modifier genes. In a broader sense, the collection of deletion suppressors of FUS toxicity is an interesting class, because these could represent attractive therapeutic targets for small molecule inhibitors or RNA interference. Taken together, the genetic modifiers uncovered by the yeast overexpression and deletion screens provide insight into the pathways affected by FUS. The way is now open to develop therapeutic strategies that target these pathways.

**Table 2:** Yeast genes that suppress or enhance FUS toxicity when deleted.

Effect	Gene	Human Homolog	Function
Suppressor	ALF1	TBCB	Alpha-tubulin folding protein
Suppressor	BUD26		Dubious open reading frame
Suppressor	CGI121	TPRKB	Protein involved in telomere uncapping and elongation
Suppressor	CLB2	CCNB1	B-type cyclin involved in cell cycle progression
Suppressor	FYV7		Essential protein required for maturation of 18S rRNA
Suppressor	GIS2	ZCCHC13	Protein proposed to be involved in the RAS/cAMP signaling pathway
Suppressor	HIT1		Protein of unknown function, required for growth at high temperature
Suppressor	HMO1		Chromatin associated high mobility group (HMG) family member involved in genome maintenance
Suppressor	IPK1		Inositol 1,3,4,5,6-pentakisphosphate 2-kinase
Suppressor	LSM7	LSM7	Lsm (Like Sm) protein, part of heteroheptameric complexes mRNA decayor in processing tRNA, snoRNA, and rRNA, involved in stress granule formation
Suppressor	LTV1		Component of the GSE complex, which is required for proper sorting of amino acid permease Gap1p
Suppressor	MFT1		Subunit of the THO complex, which is a nuclear complex involved in transcription elongation and mitotic recombination
Suppressor	MRT4	MRTO4	Protein involved in mRNA turnover and ribosome assembly, localizes to the nucleolus
Suppressor	NOP16		Constituent of 66S pre-ribosomal particles, involved in 60S ribosomal subunit biogenesis
Suppressor	NPR2	TUSC4	Component of an evolutionarily conserved Npr2/3 complex that mediates downregulation of TORC1 activity in response to amino acid limitation
Suppressor	NSR1	NCL	Nucleolar protein that binds nuclear localization sequences, required for pre-rRNA processing and ribosome biogenesis
Suppressor	NUP84	NUP107	Subunit of the nuclear pore complex (NPC), plays a role in nuclear mRNA export and NPC biogenesis
Suppressor	PPM1		Carboxyl methyltransferase, methylates the C terminus of the protein phosphatase 2A catalytic subunit
Suppressor	PUB1	TIAL1	Poly (A)+ RNA-binding protein, component of glucose deprivation induced stress granules, involved in P-body-dependent granule assembly
Suppressor	RAD50	RAD50	Subunit of MRX complex, involved in processing double-strand DNA breaks in vegetative cells
Suppressor	RPL14A	RPL14	Component of the large (60S) ribosomal subunit
Suppressor	RPL19B	RPL19	Component of the large (60S) ribosomal subunit
Suppressor	RPP2B	RPLP2	Ribosomal protein P2 beta, a component of the ribosomal stalk
Suppressor	RPS10A	RPS10L	Component of the small (40S) ribosomal subunit
Suppressor	RPS6B	RPS6	Component of the small (40S) ribosomal subunit
Suppressor	RPS8A	RPS8	Component of the small (40S) ribosomal subunit
Suppressor	SSE1	HSPA4	Hsp70 ATPase that is a component of the heat shock protein Hsp90 chaperone complex, nucleotide exchange factor for Ssa1
Suppressor	THP2		Subunit of the THO complex and TREX complex, involved in telomere maintenance
Suppressor	TSR2	TSR2	Protein with a potential role in pre-rRNA processing
Suppressor	VPS64		Protein required for cytoplasm to vacuole targeting of proteins
Suppressor	YDR417C		Dubious open reading frame, partially overlaps the verified ORF RPL12B/YDR418W
Suppressor	YGL072C		Dubious open reading frame, partially overlaps the verified gene HSF1
Suppressor	YGL088W		Dubious open reading frame, partially overlaps snR10, a snoRNA required for preRNA processing
Suppressor	YGL165C		Dubious open reading frame, partially overlaps the verified ORF CUP2/YGL166W
Suppressor	YNR005C		Dubious open reading frame
Suppressor	YOR309C	AL138690.1	Dubious open reading frame, partially overlaps the verified gene NOP58
Enhancer	ATP5	ATP50	Subunit 5 of the stator stalk of mitochondrial F1F0 ATP synthase
Enhancer	CBT1		Protein involved in 5′ end processing of mitochondrial COB, 15S_rRNA, and RPM1 transcripts
Enhancer	COX5A	COX5A	Subunit Va of cytochrome c oxidase
Enhancer	EAF1		Component of the NuA4 histone acetyltransferase complex
Enhancer	FUM1	FH	Fumarase, converts fumaric acid to L-malic acid in the TCA cycle
Enhancer	GCN4		Basic leucine zipper transcriptional activator of amino acid biosynthetic genes in response to amino acid starvation
Enhancer	KGD2	DLST	Dihydrolipoyl transsuccinylase, component of the mitochondrial alpha-ketoglutarate dehydrogenase complex
Enhancer	MAK32		Protein necessary for structural stability of L-A double-stranded RNA-containing particles
Enhancer	MRP13		Mitochondrial ribosomal protein of the small subunit
Enhancer	MRP49		Mitochondrial ribosomal protein of the large subunit, not essential for mitochondrial translation
Enhancer	MRPL39		Mitochondrial ribosomal protein of the large subunit
Enhancer	MSS1	GTPBP3	Mitochondrial protein, involved in the 5-carboxymethylaminomethyl modification of the wobble uridine base in mitochondrial tRNAs
Enhancer	OCA1		Putative protein tyrosine phosphatase, required for cell cycle arrest in response to oxidative damage of DNA
Enhancer	REC102		Protein involved in early stages of meiotic recombination
Enhancer	RIM15	MAST1	Glucose-repressible protein kinase involved in signal transduction during cell proliferation in response to nutrients
Enhancer	RTT103	RPRD1A	Protein that interacts with exonuclease Rat1p and Rai1p and plays a role in transcription termination by RNA polymerase II
Enhancer	SLM3	TRMU	tRNA-specific 2-thiouridylase, responsible for 2-thiolation of the wobble base of mitochondrial tRNAs
Enhancer	SLT2	UHMK1	Serine/threonine MAP kinase involved in regulating the maintenance of cell wall integrity and progression through the cell cycle
Enhancer	TBS1		Putative protein of unknown function
Enhancer	YDL032W		Dubious open reading frame unlikely to encode a protein, partially overlaps verified gene SLM3/YDL033C
Enhancer	YDR049W	ANKZF1	Zinc finger protein, putative transcription factor that may interact with proteins involved in histone acetylation or deacetylation
Enhancer	YDR248C	C9orf103	Putative protein of unknown function
Enhancer	YER128W		Putative protein of unknown function
Enhancer	YLR218C		Protein that localizes to the mitochondrial intermembrane space

## Discussion

We have established a pure protein aggregation assay and a yeast model to gain insight into how FUS contributes to disease pathogenesis. We have recently used a similar approach to define mechanisms underpinning TDP-43 aggregation and toxicity [14], as well as the pathogenic mechanism of ALS-linked TDP-43 mutants [15]. Using the yeast system we have also identified potent modifiers of TDP-43 toxicity [16]. One such modifier is ataxin 2, which can harbor intermediate-length polyQ expansions that are associated with increased risk for ALS in humans [16]. Like TDP-43, we find that, in isolation, FUS is an intrinsically aggregation-prone protein. FUS rapidly assembles into pore-like oligomeric species and filamentous structures that closely resemble the ultrastructure of FUS aggregates in degenerating motor neurons of ALS patients. Thus, all the information needed to assemble these structures is encoded in the primary sequence of FUS. Like TDP-43, expression of FUS in yeast results in cytoplasmic FUS aggregation, colocalization of these inclusions with stress granules and toxicity, modeling key features seen in human disease [17],[18],[21],[23],[52]. In further similarity to TDP-43, disabling the RNA binding activity of FUS reduced toxicity. Thus, we propose that the misfolded forms of FUS likely cause toxicity by binding to and sequestering essential RNAs or perhaps by interfering with the normal shuttling, stability, or metabolism of RNA. Importantly, FUS immunoreactive cytoplasmic inclusions now appear to characterize ALS and FTLD broadly, not only rare cases linked to FUS mutations [21],[23],[92]. Together these advances make it clear that FUS is a key aggregated protein in ALS, just as α-synuclein is in Parkinson's disease and huntingtin is in Huntington's disease [33].

Despite these similarities, we have uncovered key differences in the regions of the proteins that dictate aggregation and toxicity. For TDP-43, pure protein data and results from yeast and other model systems suggest that the C-terminal prion-like domain (Figure 1A) [33] plays a major role in driving aggregation [14],[15],[66],[93]. For FUS, we find that the N-terminal region, containing a predicted prion-like domain (Figure 1A) [33], is also important for aggregation in vitro and for aggregation and toxicity in yeast cells. However, C-terminal regions in FUS, particularly the first RGG domain, are also critical. Intriguingly, the first RGG domain also contains a short region (amino acids 391–407) that is detected by an algorithm designed to isolate prion-like domains [32],[33] but does not quite reach significance (Figure S1). The requirement for two specific, disparate portions of FUS for the ordered formation of filamentous structures raises the possibility that communication between the N-terminal prion-like domain (amino acids 1–239) and first RGG domain (amino acids 374–422) might mediate a self-organizing assembly process. This process might even involve an intermolecular domain swap: a common mechanism that usually involves domains at the N- and C-terminal ends of proteins and can promote the polymerization of filamentous structures in various designed and natural proteins [94]–[96]. Thus, strategies aimed at targeting either the appropriate N- or C-terminal portions of FUS could be effective at mitigating FUS aggregation in disease. Indeed, our in vitro and yeast models could open up new therapeutic avenues and provide the basic screening system to isolate specific molecules able to antagonize and reverse FUS aggregation and toxicity.

With regard to toxicity, the minimal toxic FUS fragment comprises the N-terminal prion-like domain, RRM, and the first RGG domain (1–422). These findings contrast with TDP-43, where the prion-like domain plus RRM2 are sufficient to drive aggregation and toxicity [14]. Indeed, a proteolytic fragment corresponding to these portions of TDP-43 is a pathogenic signature of ALS and FTLD-TDP [3]. By contrast, a similar pathogenic FUS fragment has not been identified in ALS or FTLD-FUS patients, which likely reflects the fact that the equivalent regions of FUS (1–373) are insufficient for aggregation and toxicity.

Mutations in the C-terminal domains of FUS and TDP-43 have both been linked to ALS [6],[37]. Interestingly, whereas some ALS-linked mutations in TDP-43 can increase stability, aggregation, cytoplasmic accumulation, and toxicity in yeast, mammalian cells, and animal models [15],[16],[78],[80],[97], the mechanisms by which FUS mutations contribute to disease appear to be distinct. Our results in yeast and with pure protein show that C-terminal FUS mutations do not promote aggregation per se. Instead of enhancing aggregation, these mutations, especially those in the extreme C-terminal region of the protein (amino acids 502–526), disrupt a NLS, leading to increased cytoplasmic accumulation of FUS [52]. Interestingly, the severity of the effects of the mutations on FUS localization in cells correlate well with age of onset of ALS in humans, with stronger mutations resulting in earlier disease onset and more cytoplasmic FUS accumulation [52]. These results suggest distinct mechanisms by which ALS-linked FUS and TDP-43 mutations contribute to disease.

Despite these differences, both TDP-43 and FUS have been shown to re-localize to stress granules and P-bodies, transient sites of RNA processing that assemble during cellular stress or injury and are conserved from yeast to man [59],[60],[62],[98]. Both TDP-43 and FUS have been purified in a complex with one another and with various components of the RNA processing machinery, including stress granules and P-bodies [62],[97]. Moreover, stress granule markers, including PABP-1, are present in disease-associated cytoplasmic FUS accumulations [52]. ALS-linked FUS mutants appear more prone to entering stress granules [51]. However, it remains unclear whether stress granule assembly contributes to FUS toxicity or is simply a downstream consequence of cellular stress associated with degeneration. Our identification of several key P-body and stress granule components as potent genetic modifiers of FUS toxicity suggests a mechanistic connection that, if validated in animal models, represents a potentially tractable new therapeutic angle. We also note that for the majority of overexpression or deletion suppressors that we have examined so far, we do not see a major difference in FUS aggregation. This suggests that these genes act downstream or in parallel to FUS aggregation. Alternatively, these modifiers may affect FUS aggregation (e.g., composition or dynamics of FUS inclusions) in subtle ways that we have so far not been able to visualize.

Curiously, there was a conspicuous lack of overlap between genetic modifiers of FUS toxicity and TDP-43 toxicity. These genetic data suggest two interesting possibilities. On one hand, targeting the modifiers in common between TDP-43 and FUS might have broad therapeutic utility for ALS. On the other hand, defining the key differences between FUS and TDP-43 pathogenic mechanisms will empower a more accurate understanding of how these seemingly similar proteins might contribute to disease in different ways.

What is the connection between TDP-43, FUS, and ALS? Does each protein contribute separately to the disease, or do they share a common disease pathway? The lack of overlap in genetic modifiers suggests that the precise mechanism of TDP-43 and FUS toxicity may be subtly different. Moreover, initial reports suggested FUS cytoplasmic accumulations were specific to rare cases of ALS, owing to FUS mutations, and that these inclusions were devoid of TDP-43 aggregates [17]. However, in one study, using optimized antigen-unmasking methods, FUS cytoplasmic immunoreactivity has recently been detected broadly in sporadic and familial ALS, including cases with TDP-43 aggregates, as well as cases without FUS mutations [92]. Further, FUS and TDP-43 have been found to physically associate in a complex [97], indicating that both TDP-43 and FUS, even in the WT state, likely contribute broadly to ALS pathogenesis. Therefore, defining mechanisms by which WT versions of these proteins are toxic to cells, as we report here for FUS and in previous studies for TDP-43 [14]–[16], will likely be informative to not only rare familial cases but to the much more common sporadic forms as well.

The discovery of RNA-binding proteins TDP-43 and FUS in ALS has re-invigorated the focus on RNA processing pathways in ALS [5],[37],[99]. Our identification of potent genetic modifiers of FUS toxicity in yeast, including a large number of conserved RNA metabolism genes, as well as key stress granule components, will provide a toehold for future studies aimed at elucidating the mechanisms by which FUS interfaces with these RNA processing pathways in disease. However, our study also suggests caution in assuming, based on sequence and structural similarity, that both TDP-43 and FUS contribute to disease via the same or similar mechanisms [38]. While there are clear similarities between the two proteins, there are also important differences, which we have defined here. Furthermore, the fact that genetic modifiers uncovered in screens for TDP-43 and FUS proteotoxicty are surprisingly distinct argues further that there are likely different underlying pathogenic mechanisms for FUS and TDP-43 proteinopathies. This conceptual framework we have established will aid the development of novel therapeutic approaches.

## Materials and Methods

### Yeast Strains, Media, and Plasmids

Yeast cells were grown in rich media (YPD) or in synthetic media lacking uracil and containing 2% glucose (SD/-Ura), raffinose (SRaf/-Ura), or galactose (SGal/-Ura).

A FUS Gateway entry clone was obtained from Invitrogen, containing full-length human FUS in the vector pDONR221. A Gateway LR reaction was used to shuttle FUS into Gateway-compatible yeast expression vectors (pAG vectors, [100], http://www.addgene.org/yeast_gateway). To generate C-terminally YFP-tagged FUS constructs, a two-step PCR protocol was used to amplify FUS (or truncated versions) without a stop codon and incorporate the Gateway attB1 and attB2 sites along with a Kozak consensus sequence. Resulting PCR products were shuttled into pDONR221 using a Gateway BR reaction. The entry clones (FUS_nostop_) were then used in LR reactions with pAG426Gal-ccdB-YFP to generate the 2 micron FUS-YFP fusion constructs and pAG416Gal-ccdB-YFP to generate the CEN FUS-YFP constructs. Primer sequences are available upon request. To generate the integrating FUS construct, the FUS entry clone was used in an LR reaction with pAG303Gal-ccdB. Expression constructs for TDP-43 have been described previously [14],[15].

ALS-linked point mutations, based on [38], were introduced into FUS using the QuickChange Site-Directed Mutagenesis Kit (Agilent) according to the manufacturer's instructions. Mutations were verified by DNA sequencing. To disable FUS RNA binding, we mutated four conserved phenylalanine residues (aa 305, 341, 359, 368) within the FUS RNA recognition motif (RRM) to leucine.

Two micron plasmid constructs (e.g., pAG426Gal-FUS-YFP) were transformed into BY4741 (*MATa his3 leu2 met15 ura3*). The FUS integrating strain was generated by linearizing pAG303Gal-FUS by Nhe I restriction digest, followed by transformation into the w303 strain (*MATa can1-100, his3-11,15, leu2-3,112, trp1-1, ura3-1, ade2-1*).

To introduce the SV40 NLS to the N-terminus of FUS, we used PCR, incorporating DNA sequences encoding the SV40 NLS (PPKKKRKV), optimized for yeast translation (CCA CCA AAA AAA AAA AGA AAA GTT) into the forward primer, following a start codon (ATG) and in frame with FUS. We verified the construct by DNA sequencing.

### Yeast Transformation and Spotting Assays

Yeast procedures were performed according to standard protocols [101]. We used the PEG/lithium acetate method to transform yeast with plasmid DNA [102]. For spotting assays, yeast cells were grown overnight at 30°C in liquid media containing raffinose (SRaf/-Ura) until they reached log or mid-log phase. Cultures were then normalized for OD600, serially diluted and spotted onto synthetic solid media containing glucose or galactose lacking uracil and were grown at 30°C for 2–3 d.

### Immunoblotting

Yeast lysates were subjected to SDS/PAGE (4%–12% gradient, Invitrogen) and transferred to a PVDF membrane (Invitrogen). Membranes were blocked with 5% nonfat dry milk in PBS for 1 h at room temperature. Primary antibody incubations were performed overnight at 4°C or at room temperature for 1–2 h. After washing with PBS, membranes were incubated with a horseradish peroxidase-conjugated secondary antibody for 1 h at room temperature, followed by washing in PBS+0.1% Tween 20 (PBST). Proteins were detected with Immobilon Western HRP Chemiluminescent Substrate (Millipore). Primary antibody dilutions were as follows: anti-GFP monoclonal antibody (Roche), 1∶5,000; Phosphoglycerate Kinase 1 (PGK1) antibody (Invitrogen), 1∶500; glyceraldehyde-3-phosphate dehydrogenase (GAPDH), 1∶5,000; FUS rabbit polyclonal antibody (Bethyl), 1∶10,000. HRP-conjugated anti-mouse and anti-rabbit secondary antibodies were used at 1∶5,000.

### Fluorescence Microscopy

For fluorescence microscopy experiments, single colony isolates of the yeast strains were grown to mid-log phase in SRaf/-Ura media at 30°C. Cultures were spun down and resuspended in the same volume of SGal/-Ura to induce expression of the FUS constructs. Cultures were induced with galactose for 4–6 h before being stained with DAPI to visualize nuclei and processed for microscopy. Images were obtained using an Olympus IX70 inverted microscope and a Photometrics CoolSnap HQ 12-bit CCD camera. Z-stacks of several fields were collected for each strain. The images were deblurred using a nearest neighbor algorithm in the Deltavision Softworx software and representative cells were chosen for figures.

### Quantification of FUS Aggregation in Yeast

To assess differences in aggregation between wild-type and mutant FUS, yeast cultures were grown, induced, and processed as described above after having normalized all yeast cultures to OD_600nm_ = 0.2 prior to galactose induction. After 6 h of induction, the identities of the samples were blinded to the observer before being examined. Several fields of cells were randomly chosen using the DAPI filter to prevent any bias towards populations of cells with increased amounts of aggregation in addition to obtaining the total number of cells in any given field. At least 200 cells per sample were counted for each replicate. Only cells with greater than three foci under the YFP channel were considered as cells with aggregating FUS.

### Yeast Plasmid Overexpression Screen

Plasmids of 5,500 full-length yeast ORFs (Yeast FLEXGene collection, [82]) were dried in individual wells of 96-well microtiter plates and transformed into a strain expressing FUS integrated at the *HIS3* locus. A standard lithium acetate transformation protocol was modified for automation and used by employing a BIOROBOT Rapidplate 96-well pipettor (Qiagen). The transformants were grown in synthetic deficient media lacking uracil (SD-Ura) with glucose. 48 h later, the cultures were inoculated into fresh SRaf-Ura media and allowed to reach stationary phase. Then the cells were spotted on to SD-Ura + glucose and SD-Ura + galactose agar plates. Suppressors and enhancers of FUS were identified on galactose plates after 2–3 d of growth at 30°C. The entire screen was repeated three times and only hits that reproduced all three times were selected for further validation. Toxicity enhancers were further tested in WT yeast cells to eliminate genes that were simply toxic when overexpressed. Immunoblotting was performed to test all modifiers for their effect on FUS expression.

### Yeast Deletion Screen

This screen was performed as described in [86],[87],[103], with some modifications, using a Singer RoToR HDA (Singer Instruments, Somerset, UK). The galactose-inducible FUS expression construct (pAG416Gal-FUS-YFP) was introduced into *MAT*α strain Y7092 (gift from C. Boone) to generate the query strain. This query strain was mated to the yeast haploid deletion collection of non-essential genes (*MAT*a, each gene deleted with KanMX cassette (confers resistance to G418)). Haploid mutants harboring the FUS expression plasmid were grown in the presence of glucose (FUS expression “off”) or galactose (FUS expression “on”). Following growth at 30°C for 2 d, plates were photographed and colony sizes measured by ImageJ image analysis software, based on [104]. The entire screen was repeated three times and only hits that reproduced all three times were selected for further validation by random spore analysis on DNA sequencing of deletion strain bar codes. Deletion strains that grew poorly on galactose were eliminated based on published data on deletion strain fitness on galactose as well as in house measurements using the yeast deletion collection.

### FUS Purification

FUS and FUS deletion mutants were expressed and purified from *Escherichia coli* as GST-tagged proteins. FUS constructs were generated in GV13 to yield a TEV protease cleavable GST-FUS protein, GST-TEV-FUS, and overexpressed in *E. coli* BL21 DE3 cells (Agilent). Protein was purified over a glutathione-sepharose column (GE) according to the manufacturer's instructions. Proteins were eluted from the glutathione sepharose with 50 mM Tris-HCl pH 8, 200 mM trehalose, and 20 mM glutathione. After purification, proteins were concentrated to 10 µM or greater using Amicon Ultra-4 centrifugal filter units (10 kDa molecular weight cut-off; Millipore). Protein was then centrifuged for 30 min at 16,100 g to remove any aggregated material. After centrifugation, the protein concentration was determined by Bradford assay (Bio-Rad) and the proteins were used immediately for aggregation reactions. GST-TEV-TDP-43 was purified as described [15].

### FUS-RNA binding Assay

RNA-binding assays were performed as described [105]. Briefly, FUS RNA probe was transcribed by T7 polymerase from DNA template (5′-GTAATACGACTCACTATAGGGGAAAATTAATGTGTGTGTGTGGAAAATT-3′) with ^32^P-labeled UTP. Probes were gel-purified and adjusted to 10^4^ c.p.m./µl specific activity. Standard binding reactions were carried out in 10 µl, with a final concentration of 4 mM MgCl_2_, 25 mM phosphocreatine, 1.25 mM ATP, 1.3% polyvinyl alcohol, 25 ng of yeast tRNA, 0.8 mg of BSA, 1 mM DTT, 0.1 µl Rnasin (Promega, 40 U/ml), 75 mM KCl, 10 mM Tris, pH 7.5, 0.1 mM EDTA, 10% glycerol, and 0.15 µM to 5 µM GST-FUS or GST. Binding reactions were incubated for 20 min at 30°C with ^32^P-labeled probe. After binding, heparin was added to a final concentration of 0.5 µg/ml; reactions were analyzed on a 4.5% native gel (Acrylamide/Bis 29:1, BioRad).

### FUS In Vitro Aggregation Assay

Aggregation was initiated by the addition of TEV protease (Invitrogen) to GST-TEV-FUS (2.5–5 µM) in assembly buffer (AB): 100 mM TrisHCl pH 8, 200 mM trehalose, 0.5 mM EDTA, and 20 mM glutathione. Aggregation reactions were incubated at 22°C for 0–90 min with or without agitation at 700 rpm in an Eppendorf Thermomixer. No aggregation occurred unless TEV protease was added to separate GST from FUS or TDP-43. Turbidity was used to assess aggregation by measuring absorbance at 395 nm. For sedimentation analysis, reactions were centrifuged at 16,100 g for 10 min at 25°C. Supernatant and pellet fractions were then resolved by SDS-PAGE and stained with Coomassie Brilliant Blue, and the amount in either fraction determined by densitometry in comparison to known quantities of FUS. For electron microscopy (EM) of in vitro aggregation reactions, protein samples (20 µl of a 2.5 µM solution) were adsorbed onto glow-discharged 300-mesh Formvar/carboncoated copper grid (Electron Microscopy Sciences) and stained with 2% (w/v) aqueous uranyl acetate. Excess liquid was removed, and grids were allowed to air dry. Samples were viewed using a JEOL 1010 transmission electron microscope.

### Visualizing P-Bodies and Stress Granules in Yeast

We used fluorescent markers of P-bodies and stress granules and live cell imaging to monitor stress granule and P-body formation in yeast, based on standard protocols [63]. First, we transformed yeast strain BY4741 with pAG423GAL-FUS-YFP. This strain was then transformed with plasmids encoding P-body markers (Lsm1-mCherry, LEU2 or Dcp2-RFP, LEU2) or stress granule markers (Pub1-RFP, URA3 or CFP-Pbp1, URA3) separately. Transformants were grown overnight to mid-log phase in raffinose-containing media. To induce expression of FUS-YFP, galactose was added to 2% and cells were incubated at 30°C for 4 h and then processed for microscopy. We used a spinning disk confocal microscope to monitor the YFP, CFP, and RFP signals in live cells. For each channel, 60 z-sections were acquired at 0.1 µm increments at 23°C. Figures display the maximum projection of each channel.

### FUS and Modifier Genes Transfection in Mammalian Cells

HEK293T cells were plated in 96-well format and transfected with FuGene (Roche) according to the manufacturer's instructions. 72 h post-transfection, MTT (3-(4,5-Dimethylthiazol-2-yl)-2,5-diphenyltetrazolium bromide) (Sigma) was added to each well and incubated for 3 h at 37°C. Acidic Isoproponal (40 mM HCl) was then added to each well to solubilize the blue formazan crystals. Absorbance of each well was read with a Tecan Safire II plate reader using 570 nm for absorbance and 630 nm as a reference wavelength. Absorbance measurements were normalized to the absorbance of untransfected cells and used to calculate a percent viability for each condition.

## Supporting Information

Figure S1 Prion domain prediction algorithm identifies prion-like domains in TDP-43 (top) and FUS/TLS (bottom). Note that the prion-like domain (PrD) of TDP-43 is located in the C-terminal region, whereas the PrD of FUS/TLS is in the N-terminal region. There is an additional peak of PrD character predicted by the algorithm in FUS/TLS aa 391–407. For additional details on design and implementation of this prion domain prediction algorithm, see [33],[34].
(3.34 MB TIF)

Figure S2 FUS and TDP-43 co-localize in yeast cells. FUS-YFP and TDP-43-CFP were co-transformed into yeast cells and their localization visualized by fluorescence microscopy. FUS-YFP and TDP-43-CFP co-localized to the same subcellular foci (arrows).
(1.46 MB TIF)

Figure S3 FUS localizes to the nucleus and cytoplasm when expressed at lower levels. Yeast strain YEF6030 (YEF473a NUP57-mCherry-His3), harboring a nuclear envelope marker, to visualize the nucleus in live cells, was transformed with 416GPD-FUS-YFP. FUS localization in live cells was visualized using a spinning disc confocal microscope. At this level of expression, FUS-YFP localized to the nucleus (arrows) and cytoplasm in a diffuse pattern.
(1.98 MB TIF)

Figure S4 FUS truncation proteins localize to the nucleus. DAPI stained cells confirm nuclear localization of FUS truncation constructs 1–168aa, 1–269aa, and 1–373aa (also see Figure 3 of main text).
(4.59 MB TIF)

Figure S5 Verifying FUS toxicity modifiers from plasmid overexpression screen. Spotting assay showing serial dilutions of yeast cells expressing FUS along with empty vector control, four enhancers, or six suppressors from the screen.
(4.44 MB TIF)

## Abbreviations

ALS: amyotrophic lateral sclerosis
EM: electron microscopy
FTLD: frontotemporal lobar degeneration
FUS: fused in sarcoma
GPD: glyceraldehyde-3-phosphate dehydrogenase
GST: glutathione-S-transferase
NLS: nuclear localization signal
PABP: polyA-binding protein
P-bodies: processing bodies
PrD: prion-like domain
PrP: prion protein
RRM: RNA recognition motif
SCA: spinocerebellar ataxia
SGA: synthetic genetic array
TDP: TAR-DNA-binding protein
TEV: tobacco etch virus
Tif: translation initiation factor
TLS: translocated in liposarcoma
WT: wild-type
YFP: yellow fluorescent protein
