# Supplementary figures and images for: Molecular Determinants and Genetic Modifiers of Aggregation and Toxicity for the ALS Disease Protein FUS/TLS
Source: PLoS Biol. 2011 Apr 26;9(4):e1000614. doi: 10.1371/journal.pbio.1000614 (PMC3082519; doi:10.1371/journal.pbio.1000614)

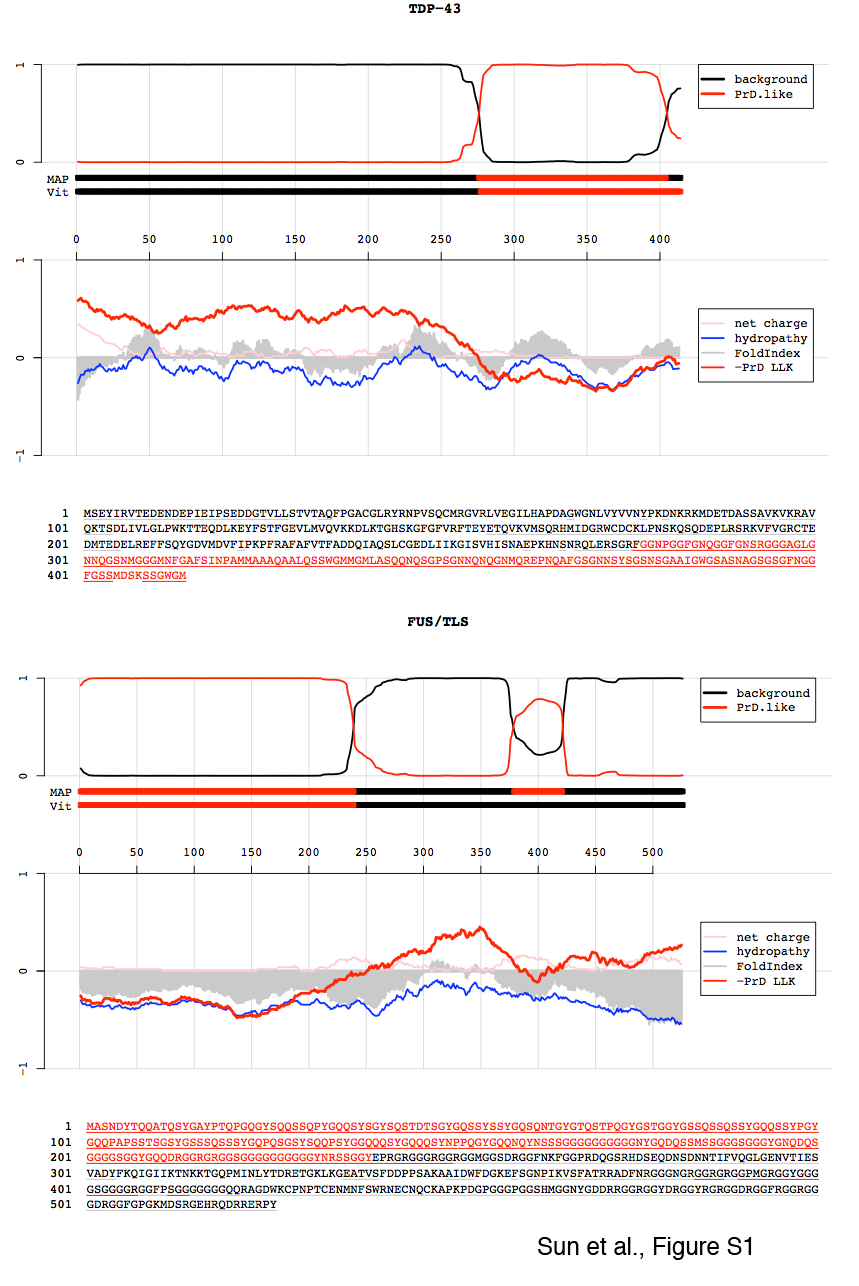

Supplement: Figure S1 — Prion domain prediction algorithm identifies prion-like domains in TDP-43 (top) and FUS/TLS (bottom). Note that the prion-like domain (PrD) of TDP-43 is located in the C-terminal region, whereas the PrD of FUS/TLS is in the N-terminal region. There is an additional peak of PrD character predicted by the algorithm in FUS/TLS aa 391–407. For additional details on design and implementation of this prion domain prediction algorithm, see [33],[34]. (3.34 MB TIF) [file pbio.1000614.s001.tif]

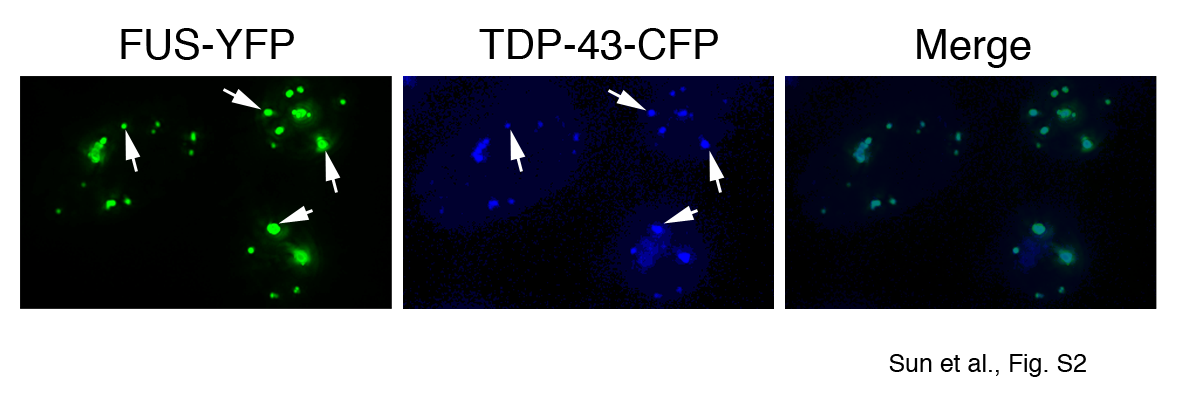

Supplement: Figure S2 — FUS and TDP-43 co-localize in yeast cells. FUS-YFP and TDP-43-CFP were co-transformed into yeast cells and their localization visualized by fluorescence microscopy. FUS-YFP and TDP-43-CFP co-localized to the same subcellular foci (arrows). (1.46 MB TIF) [file pbio.1000614.s002.tif]

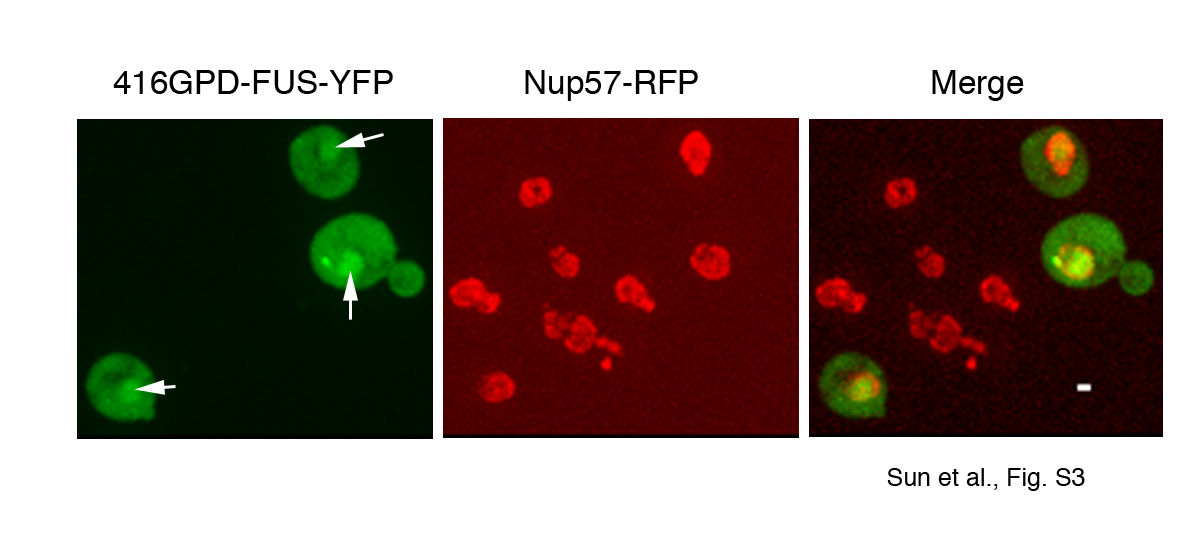

Supplement: Figure S3 — FUS localizes to the nucleus and cytoplasm when expressed at lower levels. Yeast strain YEF6030 (YEF473a NUP57-mCherry-His3), harboring a nuclear envelope marker, to visualize the nucleus in live cells, was transformed with 416GPD-FUS-YFP. FUS localization in live cells was visualized using a spinning disc confocal microscope. At this level of expression, FUS-YFP localized to the nucleus (arrows) and cytoplasm in a diffuse pattern. (1.98 MB TIF) [file pbio.1000614.s003.tif]

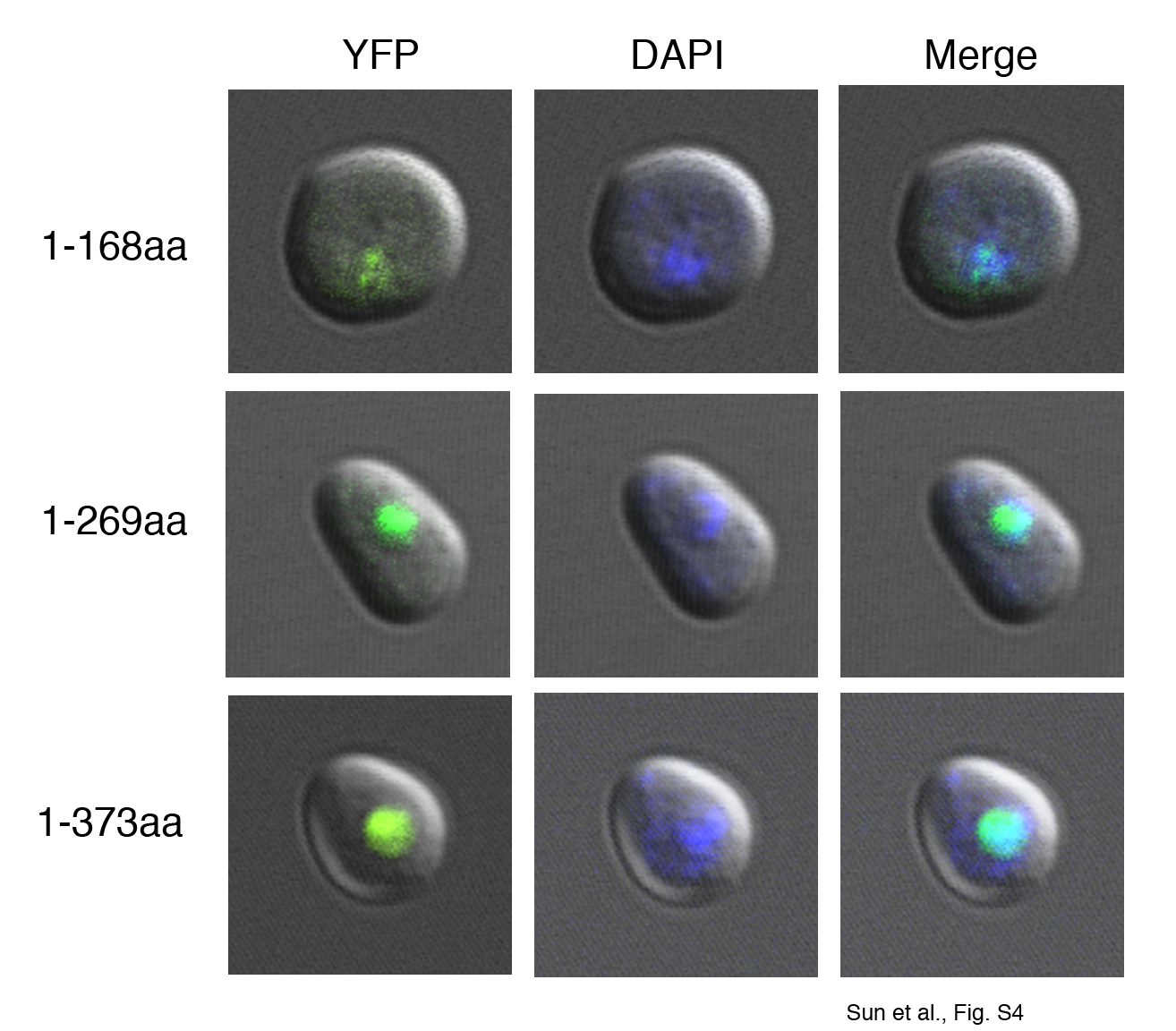

Supplement: Figure S4 — FUS truncation proteins localize to the nucleus. DAPI stained cells confirm nuclear localization of FUS truncation constructs 1–168aa, 1–269aa, and 1–373aa (also see Figure 3 of main text). (4.59 MB TIF) [file pbio.1000614.s004.tif]

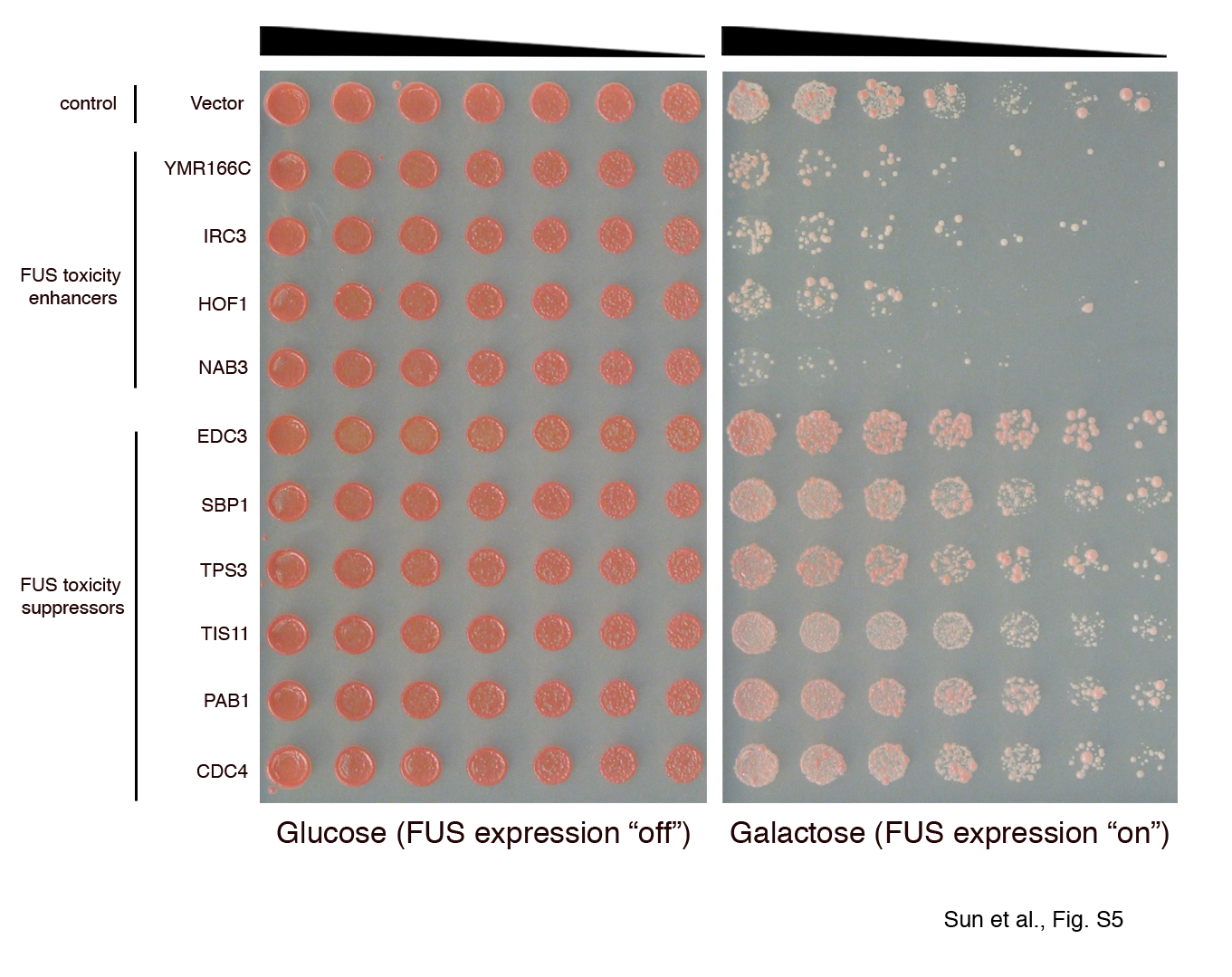

Supplement: Figure S5 — Verifying FUS toxicity modifiers from plasmid overexpression screen. Spotting assay showing serial dilutions of yeast cells expressing FUS along with empty vector control, four enhancers, or six suppressors from the screen. (4.44 MB TIF) [file pbio.1000614.s005.tif]
